# Safety and efficacy of probiotic supplementation in 8 types of inflammatory arthritis: A systematic review and meta-analysis of 34 randomized controlled trials

**DOI:** 10.3389/fimmu.2022.961325

**Published:** 2022-09-23

**Authors:** Liuting Zeng, Ying Deng, Qi He, Kailin Yang, Jun Li, Wang Xiang, Huiping Liu, Xiaofei Zhu, Hua Chen

**Affiliations:** ^1^ Department of Rheumatology and Clinical Immunology, Peking Union Medical College Hospital, Chinese Academy of Medical Sciences & Peking Union Medical College, National Clinical Research Center for Dermatologic and Immunologic Diseases (NCRC-DID), Key Laboratory of Rheumatology and Clinical Immunology, Ministry of Education, Beijing, China; ^2^ People’s Hospital of Ningxiang City, Ningxiang, China; ^3^ Key Laboratory of Hunan Province for Integrated Traditional Chinese and Western Medicine on Prevention and Treatment of Cardio-Cerebral Diseases, Hunan University of Chinese Medicine, Changsha, China; ^4^ The First People's Hospital of Changde City, Changde, China; ^5^ Fudan University, Shanghai, China

**Keywords:** inflammatory arthritis, hyperuricemia and gout, inflammatory bowel disease arthritis, juvenile idiopathic arthritis, osteoarthritis, osteoporosis and osteopenia, spondyloarthritis, probiotics

## Abstract

**Objective:**

To evaluate Safety and efficacy of probiotic supplementation in inflammatory arthritis.

**Methods:**

The literature on the treatment of inflammatory arthritis with probiotics has been collected in databases such as CNKI, Pubmed, Cochrane library, Embase, etc. The search time is for them to build the database until May 2022. The included literatures are randomized controlled trials (RCTs) of probiotics in the treatment of hyperuricemia and gout. The Cochrane risk assessment tool was used for quality evaluation, and the Rev Man5.3 software was used for meta-analysis.

**Results:**

A total of 37 records were finally included, involving 34 RCTs and 8 types of autoimmune disease (Hyperuricemia and gout, Inflammatory bowel disease arthritis, juvenile idiopathic arthritis [JIA], Osteoarthritis [OA], Osteoporosis and Osteopenia, Psoriasis, rheumatoid arthritis (RA), Spondyloarthritis). RA involved 10 RCTs (632 participants) whose results showed that probiotic intervention reduced CRP. Psoriasis involved 4 RCTs (214 participants) whose results showed that probiotic intervention could reduce PASI scores. Spondyloarthritis involved 2 RCTs (197 participants) whose results showed that probiotic intervention improved symptoms in patients. Osteoporosis and Ostepenia involving 10 RCTs (1156 participants) showed that probiotic intervention improved bone mineral density in patients. Hyperuricemia and gout involving 4 RCTs (294 participants) showed that probiotic intervention improved serum uric acid in patients. OA involving 1 RCTs (433 participants) showed that probiotic intervention improved symptoms in patients. JIA involving 2 RCTs (72 participants) showed that probiotic intervention improved symptoms in patients. Inflammatory bowel disease arthritis involving 1 RCTs (120 participants) showed that probiotic intervention improved symptoms in patients. All of the above RCTs showed that probiotics did not increase the incidence of adverse events.

**Conclusion:**

Probiotic supplements may improve Hyperuricemia and gout, Inflammatory bowel disease arthritis, JIA, OA, Osteoporosis and Osteopenia, Psoriasis, RA, Spondyloarthritis. However, more randomized controlled trials are needed in the future to determine the efficacy and optimal dosing design of probiotics.

**Systematic Review Registration:**

https://www.crd.york.ac.uk/prospero/display_record.php?ID=CRD42021286425, identifier CRD42021286425.

## 1 Introduction

Chronic inflammatory arthritis disease is a multifactorial disease characterized by autoantibody production and systemic features. The main pathological features of inflammatory arthritis are persistent synovitis and joint erosion, in which synovitis induces the pannus and joint destruction of arthritis (1). Chronic inflammatory arthritis, as one of the important clinical manifestations of rheumatic immune diseases, often leads to joint pain, limited mobility, and eventually joint deformity and disability (2, 3). The main clinical features of chronic inflammatory arthritis are chronic progression and recurrent attacks, which cause a huge burden of disease and pain in patients. There are many types of inflammatory arthritis, mainly include: (1) rheumatoid arthritis (RA) (4); (2) Gout (gout is characterized by a buildup of uric acid that forms crystals in the joints—especially in the big toe, and sometimes in the hands, wrists, or knees) (5); (3) psoriatic arthritis (about 30 percent of people with psoriasis (an autoimmune disease that causes scaly raised skin bumps) develop psoriatic arthritis, which affects the knees, ankles, wrists, or fingers) (6); (7) osteoarthritis (OA) (8); (4) ankylosing spondylitis (9); (6) arthritis associated with inflammatory bowel disease (10). Among them, epidemiological surveys show that globally, the prevalence of RA is estimated to be 0.24%. Currently, the total number of RA patients in the top 10 global drug markets exceeds 7 million, and this number will exceed 8.5 million by 2023 (11). Gouty arthritis caused by hyperuricemia has also increased rapidly, becoming one of the most common types of arthritis (12, 13). Current treatment drugs mainly include: (1) Non-steroidal anti-inflammatory drugs (NSAIDs) (which reduce the level of prostaglandins – chemicals that promote inflammation) (14). (2) Steroid hormones (which reduce inflammation and suppress the immune system) (15). (3) Anti-rheumatic drugs (DMARDs) (16). (4) Drugs that lower uric acid levels (gout) (17). (5) Other related nutritional supplements. However, the above-mentioned drug-related adverse reactions are common and cannot guarantee remission, and are prone to drug resistance over time leading to treatment failure (18–20). Therefore, there is a need for new related target therapeutic approaches for drug development and treatment of joint inflammation, thereby reducing the disease burden of inflammatory arthritis. A study showed that gut microbial dysbiosis (in combination with environmental triggers) may contribute to inflammatory immune disturbances in inflammatory arthritis in combination with genetically predisposed individuals (21). As shown in a study of a subset of patients with early RA, subclinical intestinal inflammation was present in almost all patients (22). Intestinal inflammation in these patients was characterized by increased numbers of infiltrating monocytes, T cells, B cells, and CD68+ macrophages, as well as the presence of lymphoid follicles. These histological findings suggest that a chronic inflammatory process develops in the gut of patients with early RA (23). Furthermore, in the presence of inflammation, alterations in gut barrier function and concomitant increases in gut permeability and bacterial translocation can promote inflammatory bowel disease and autoimmune responses in genetically susceptible hosts (24). Signs of altered intestinal permeability have been observed in RA patients, which may be related to the possible entry of inflammatory immune cells in the intestinal tissue into joints (24).

Probiotics are defined by the Food and Agriculture Organization of the United Nations (FAO) and the World Health Organization (WHO) as “ live microorganisms that, when administered in adequate amounts, confer a health benefit on the host” (25). Numerous *in vivo* and *in vitro* studies have shown that probiotics can exert immunomodulatory effects in several ways: (1) regulate intestinal inflammation and immune function; (2) prevent the increase in intestinal permeability and bacterial translocation that accompanies disruption of intestinal barrier function; (3) reduce the production of autoantibodies in the inflamed intestine; (4) reduce the entry of pro-inflammatory immune cells in the gut tissue into the joints (26). For example, the genera *Lactobacillus* and *Bifidobacterium* contain strains with anti-inflammatory properties and are important probiotics. It has been proven that some strains of *L. casei* can alleviate RA in rats by increasing the body’s anti-inflammatory cytokines (such as IL-10, TGF-β) and inhibiting pro-inflammatory cytokines (such as IL-1β, IL-2, IL-6, IL-12, IL-17) (27). Both *L. plantarum* LC27 and *B. longum* LC67 can inhibit the inflammatory response by inhibiting the nuclear factor kappa-B (NF-κB) inflammatory pathway (28). *L. casei* can promote the differentiation of CD4+ T cells into regulatory T cells (Treg) and inhibit their differentiation into Th17 cells, enhance the function of Treg cells, inhibit the function of Th17, and relieve inflammatory arthritis through immune regulation (29). A number of randomized controlled trials (RCTs) have also shown that probiotics can inhibit inflammatory factors, regulate immunity and improve pain scores in the intervention of arthritis. However, due to different types of probiotics and different types of arthritis, RCTs need to be summarized and summarized to clarify their clinical efficacy and safety. Therefore, this systematic review and meta-analysis summarizes and analyzes the RCTs of probiotics in the treatment of inflammatory arthritis so as to provide reference information for clinical application.

## 2 Materials and methods

### 2.1 Protocol

This systematic review and meta-analysis were conducted strictly in accordance with the protocol registered in PROSPERO (CRD42021286425) and PRISMA-guidelines (see **Supplementary Material**).

### 2.2 Search criteria

#### 2.2.1 Inclusion criteria

(1) Study type: The included studies were RCTs, with no restrictions on random sequence generation methods, and no restrictions on language and publication time. (2) Participants: patients diagnosed with a type of inflammatory arthritis according to accepted criteria. (3) Intervention methods: The experimental group was an intervention containing a probiotic preparation (preparation type, dose, and type of probiotics are not limited). The control group was the intervention without the probiotic preparation. (4) Outcomes: Disease efficacy indicators, inflammatory indicators and adverse events.

#### 2.2.2 Exclusion criteria

(1) Non-RCT; (2) Animal experiments, reviews, etc.; (3) Unable to view full text for data extraction; (4) The therapy of the control group included probiotics.

### 2.3 Search databases

Web of Science, China National Knowledge Infrastructure (CNKI), VIP Database for Chinese Technical Periodicals, Sinomed, Pubmed, Embase, Wanfang Database on Academic Institutions in China, Medline Complete, ClinicalTrials.gov and Cochrane Library were searched from the time of their establishment to May 15, 2022. The search strategy was shown in **Table S1**.

### 2.4 Search strategy, data extraction and quality assessments

Two researchers independently screened the literature and extract the data according to the established inclusion and exclusion criteria. The literature was initially screened by reading the title and abstract, and those that obviously did not meet the inclusion criteria were excluded. Subsequently, the remaining articles were read in full to determine their final inclusion. In case of disagreement, it was decided by discussion with all researchers.

Regarding literature quality assessment, the final included RCTs were assessed for randomization, allocation concealment, blinding, incompleteness of outcomes, selective reporting, and other risks using risk of bias assessment tools by 2 researchers (30). In case of disagreement, it was decided by discussion with all researchers.

### 2.5 Statistical analysis

This study used RevMan 5.3 software provided by the Cochrane Collaboration for statistical analysis (31). For continuous variables, mean difference (MD) was used as the pooled effect size. Heterogeneity test was performed for each analysis. If I2≥50%, P<0.1, indicating the existence of heterogeneity, the random-effects model was used for analysis; if it was homogeneous, the fixed-effects model was used for analysis.

## 3 Results

### 3.1 Literature search results

**Figure 1:**
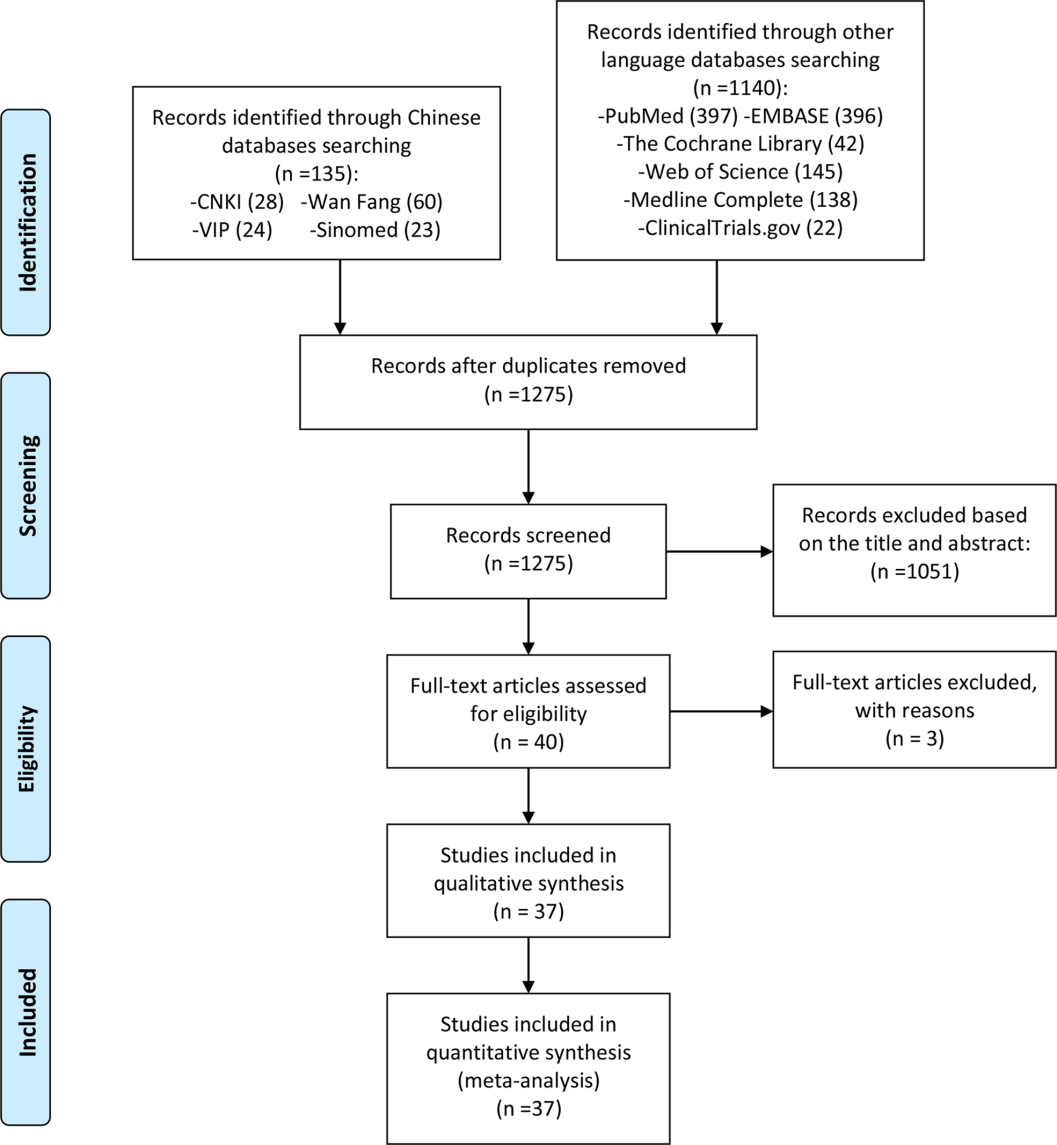
Flow diagram of clinical research.

A total of 40 relevant studies were obtained in the initial examination, and after screening, 37 records were finally included (32–68), and 3 records were excluded for they are not RCTs (69–71). The literature screening process and results are shown in **Figure 1**.

### 3.2 Description of included trials

Three records (32–34) came from the same RCT and were therefore recorded as Alipour et al., 2014 (32–34). Two records (56, 57) came from the same RCT and were therefore recorded as Nilsson et al., 2018 (56, 57). The included RCTs involved 8 inflammatory arthritis (Hyperuricemia and gout, Inflammatory bowel disease arthritis, JIA, OA, Osteoporosis and Osteopenia, Psoriasis, RA, Spondyloarthritis) and were from 14 different countries (Iran, Finland, the U.S.A., Canada, Sweden, China, Brazil, Spanish, Ireland, New Zealand, the U.K., Denmark, Japan, India). The details of study characteristics are presented in **Table 1**.

**Table 1:** The characteristics of the included studies.

Disease	Study	Trial registration number	Country	Sample size		Intervention		Relevant outcomes	Mean age (years)		Duration
				Trial group	Control group	Trial group	Control group		Trial group	Control group	
RA	Alipour et al., 2014 (32–34)	IRCT201206234105N9	Iran	24	22	*L. casei* 1×10^8^ CFU	Placebo	DAS28, CRP, Number of tender joints, Number of swollen joint, adverse events	41.14 ± 12.65	44.29 ± 9.77	8 weeks
	Hatakka et al., 2003 (35)	–	Finland	8	13	*L. rhamnosus* GG 1×10^10^ CFU	Placebo	CRP, ESR, Number of tender joints, Number of swollen joint	50 ± 10	53 ± 7	12 months
	Mandel et al., 2010 (36)	ACTRN12609000435280	the U.S.A.	22	22	*Bacillus coagulans* GBI-30, 6086 2×10^8^ CFU	Placebo	CRP, ESR, Number of tender joints, Number of swollen joint, adverse events	36-82		2 weeks
	Pineda Mde et al., 2011 (37)	Not provide	Canada	15	14	*L. rhamnosus* GR-1 4×10^8^ CFU + *L. reuteri* RC-1 4×10^8^ CFU	Placebo	DAS28, CRP, ESR, Number of tender joints, Number of swollen joint, adverse events	63.8 ± 7.5	59.1 ± 9.1	12 weeks
	Zamani et al., 2016 (38)	IRCT201511015623N58	Iran	30	30	*L. acidophilus* 2×10^9^ CFU + *L. casei* 2×10^9^ CFU + *B. bifidum* 2×10^9^ CFU	Placebo	DAS28, CRP, Number of tender joints, Number of swollen joint	52.2 ± 12.2	50.6 ± 13.1	8 weeks
	Zamani et al., 2017 (39)	IRCT201611165623N94	Iran	27	27	*L. acidophilus* 2×10^9^ CFU + *L. casei* 2×10^9^ CFU + *B. bifidum* 2×10^9^ CFU	Placebo	DAS28, CRP	49.5 ± 12.9	49.3 ± 11.0	8 weeks
	Esmaeili et al., 2020 (40)	IRCT20121216011763N37	Iran	186		*L. acidophilus* 2×10^9^ CFU + *L. bulgaricus* 2×10^9^ CFU + *L. casei* 2×10^9^ CFU + *L. rhamnosus* 2×10^9^ CFU + *B. breve* 2×10^9^ CFU + *B. longum* 2×10^9^ CFU + *Streptococcus thermophiles* 2×10^9^ CFU + Methotrexate 15-20 mg + Prednisolone 0-2.5 mg	Placebo + Methotrexate 15-20 mg + Prednisolone 0-2.5 mg	DAS28, CRP, ESR, Number of tender joints, Number of swollen joint	17-85		12 weeks
	Vadell et al., 2020 (41)	NCT02941055	Sweden	26	24	Anti-inflammatory foods (including *L. plantarum* 299v)	General dietary intake in Sweden (without probiotics)	DAS28, CRP, ESR, Number of tender joints, Number of swollen joint, adverse events	61 ± 12		10 weeks
	Gao et al., 2017 (42)	–	China	50	50	*B. infantis*, *L. acidophilus* and *Enterococcus faecalis*≥4.5×10^6^ CFU; *Bacillus cereus*≥4.5×10^5^CFU+ Loxoprofen sodium 60 mg T.i.d + methotrexate 10 mg once a week, leflunomide 10 mg Q.d.	Loxoprofen sodium 60 mg T.i.d + methotrexate 10 mg once a week, leflunomide 10 mg Q.d.	DAS28, CRP, ESR, Number of tender joints, Number of swollen joint, adverse events	43.88 ± 7.34	44.50 ± 7.55	12 weeks
	Cannarella et al., 2021 (43)	–	Brazil	21	21	*L. acidophilus* La-14 2×10^9^ CFU + *L. casei* Lc-11 2×10^9^ CFU + *Lactococcus lactis* Ll-23 2×10^9^ CFU + *B. lactis* Bl-04 2×10^9^ CFU + *B. bifidum* Bb-06 + maltodextrin	Placebo + maltodextrin	DAS28, CRP, ESR	48-64	49-68	8 weeks
Psoriasis	Navarro-López et al., 2019 (44)	NCT02576197	Spanish	45	43	*B. longum* CECT 7347, *B. lactis* CECT 8145 and *L. rhamnosus* CECT 8361≥1×10^9^ CFU + Topical corticosteroid betamethasone in combination with calcipotriol	Oral placebo (maltodextrin)+Topical corticosteroid betamethasone in combination with calcipotriol	PASI score, adverse events	41.57 ± 13.23	43.09 ± 10.32	12 weeks
	Groeger et al., 2013 (45)	–	Ireland	12	14	*B. infantis* 35264 1×10^10^ CFU	Oral placebo (maltodextran)	CRP, TNF-α and IL-6	–	–	8 weeks
	Lu 2017 (46)	–	China	25	25	*B. infantis*, *L. acidophilus* and *Enterococcus faecalis*≥4.5×10^6^ CFU; *Bacillus cereus*≥4.5×10^5^ CFU+Oral Acitretin 10mg Tid	Oral Acitretin 10mg Tid	PASI score	51.3 ± 5.6	52.2 ± 5.9	12 weeks
	Moludi et al., 2021 (47)	IRCT20180712040438N2	Iran	25	25	*L. acidophilus* 3.6×10^9^ CFU + *B. bifidum* 3.6×10^9^ CFU + *B. lactis* 3.6×10^9^ CFU + *B. langum* 3.6×10^9^ CFU	Placebo + maltodextrin	PASI score, CRP, TNF-α and IL-6, adverse events	42.70 ± 9.10	43.10 ± 7.80	8 weeks
Spondyloarthritis	Jenks et al., 2010 (48)	–	New Zealand	32	31	*Streptococcus salivarius* K12 1.6×10^8^ CFU + *B. lactis* LAFTI B94 6.4×10^8^ CFU + *L. acidophilus* LAFTI L10 6.4×10^8^ CFU	Placebo	Efficacy indicators, adverse events	45.5 ± 15	41.1 ± 10	12 weeks
	Brophy et al., 2008 (49)	ISRCTN36133252	the U.K.	69	65	*L. salivarius* (CUL61) 6.25×10^9^ CFU + *L. paracasei* (CUL08) 1.25×10^9^ CFU + *B. infantis* (CUL34) 1.25×10^9^ CFU + *B. bifidum* (CUL20) 1.25×10^9^ CFU	Placebo	Efficacy indicators, adverse events	44.8 ± 12.1	42.7 ± 12.7	12 weeks
Osteoporosis and Osteopenia	Guo et al., 2020 (50)	–	China	30	24	*B. lactis* Probio-M8 1.5×10^10^ CFU	Placebo	BMD	61.91 ± 6.37	6.34 ± 5.71	6 months
	Jafarnejad et al., 2017 (51)	IRCT2015092024103N1	Iran	20	21	*L. casei* 1.3×10^10^ CFU + *B. longum* 5 ×10^10^ CFU + *L. acidophilus* 1.5×10^10^ CFU + *L. rhamnosus* 3.5×10^9^ CFU + *L. bulgaricus* 2.5 ×10^8^ CFU + *B. breve* 1×10^10^ CFU + *Streptococcus thermophilus* 1.5×10^8^ CFU	Placebo	BMD	58.85 ± 0.68	57.29 ± 0.72	6 months
	Jansson et al., 2019 (52)	NCT02722980	Sweden	126	123	*L. paracasei* 8700:2 (DSM 13434) + *L. plantarum* Heal 9 (DSM 15312) + *L. plantarum* Heal 19 (DSM 15313) total 1*10^10^ CFU	Placebo	BMD, Adverse events	59.1 ± 3.8	58.1 ± 4.3	12 months
	Lambert et al., 2017 (53)	NCT02174666	Denmark	38	40	Red clover extract (RCE) (rich in isoflavone aglycones and probiotics)	Placebo [made by 90 L of water mixed with 250 g brown food coloring (ammoniated caramel) (Kavli)]	BMD, adverse events	60.84 ± 1.07	62.85 ± 0.99	12 months
	Li et al., 2021 (54)	–	China	73	73	*B. infantis*, *L. acidophilus* and *Enterococcus faecalis*≥4.5×10^6^ CFU; *Bacillus cereus*≥4.5×10^5^CFU + Oral alendronate sodium + subcutaneous or intramuscular injection of salmon calcitonin	Oral alendronate sodium 10 mg Qd + subcutaneous or intramuscular injection of salmon calcitonin 50 IU Qd.	BMD, Adverse events	68. 15 ± 22.36	69. 82 ± 21.47	6 months
	Liu 2019 (55)	–	China	42	45	*B. longum*, *L. acidophilus* and *Enterococcus faecalis*≥2×10^7^ CFU + Conventional therapy	Conventional therapy	BMD	70.5 ± 6.8	69.8 ± 6.4	6 months
	Nilsson et al., 2018 (56, 57)	NCT02422082	Sweden	45	45	Freeze-dried *L. reuteri* 6475 1*10^10^ CFU	Placebo (maltodextrin powder)	BMD, Adverse events	76.4 ± 1.0	76.3 ± 1.1	12 months
	Song et al., 2020 (58)	–	China	100	100	*B. infantis*, *L. acidophilus* and *Enterococcus faecalis*≥4.5×10^6^ CFU; *Bacillus cereus*≥4.5×10^5^CFU + Conventional therapy	Conventional therapy	BMD	68. 20 ± 12. 78	69. 76 ± 12. 09	12 months
	Takimoto et al., 2018 (59)	–	Japan	31	30	Probiotic *Bacillus subtilis* C-3102 (C-3102) 3.4*10^9^ CFU	Placebo	BMD, Adverse events	57.5 ± 4.3	57.8 ± 5.4	6 months
	Wang et al., 2019 (60)	–	China	75	75	*B. longum*, *L. acidophilus* and *Enterococcus faecalis*≥2×10^7^ CFU + Conventional therapy	Conventional therapy	BMD	71.52 ± 5.46	71.68 ± 5.41	2 months
Hyperuricemia and gout	Yamanaka et al., 2019 (61)	UMIN000021837	Japan	13	12	PA-3Y, yogurt containing *L. delbrueckii* ssp. *bulgaricus* and *Streptococcus thermophilus* (PA-3) at 8.5×10^7^cfu/g or more (100g)	Yogurt beverage (100g) without PA-3	Serum uric acid levels	63.0 ± 8.5	63.6 ± 6.9	8 weeks
	Kamatani et al., 2018 (62)	–	Japan	40	20	PA-3 3×10^7^ CFU/g (85g); PA-3 3×10^6^CFU/g (85g)	Yogurt beverage (85g) without PA-3	Serum uric acid levels	>35		8 weeks
	Zhan et al., 2020 (63)	–	China	50	50	*Clostridium butyricum* ≥ 0.735×10^6^ CFU + febuxostat	Febuxostat	Serum uric acid levels	18-75		4 weeks
	Wang and Xu 2022 (64)	–	China	55	54	*B. longum*, *L. acidophilus* and *Enterococcus faecalis*≥2×10^7^ CFU + febuxostat	Febuxostat	Serum uric acid levels	54. 08 ± 3. 99	52. 41 ± 4. 67	8 weeks
OA	Lei et al., 2017 (65)	–	China	215	218	*L. casei* Shirota ≥ 6×10^9^ CFU	Placebo	Efficacy indicators, adverse events	66.5 ± 5.2	67.2 ± 4.8	6 months
JIA	Shukla et al., 2016 (66)	CTRI/2012/08/002871	India	22	20	VSL3 (containing *Streptococcus thermophilus*, *B. breve*, *B. longum*, *B. infantis*, *L. acidophilus*, *L. plantarum*, *L. paracasei*, and *L. delbrueckii* subsp. *bulgaricus*.) 2.5×10^11^ CFU	Placebo	Efficacy indicators	15 ± 2.5		12 weeks
	Malin et al., 1997 (67)	–	Finland	10	20	*L. rhamnosus* GG 2×10^10^ CFU	Colostrum or Immune colostrum	Efficacy indicators	1-15		2 weeks
Inflammatory bowel disease arthritis	Zhang et al., 2020 (68)	–	China	60	60	A: Probiotic capsules (contains *B. longum*, *L. acidophilus*); B: Probiotic capsules + Narrative Medical Education	A: Routine health education and treatment guidance; B: Narrative Medical Education only	Efficacy indicators	A: 36.46 ± 4.22; B: 37.41 ± 5.88	A: 35.48 ± 4.96; B: 37.22 ± 5.34	8 weeks

### 3.3 Risk of bias assessments

**Figure 2:**
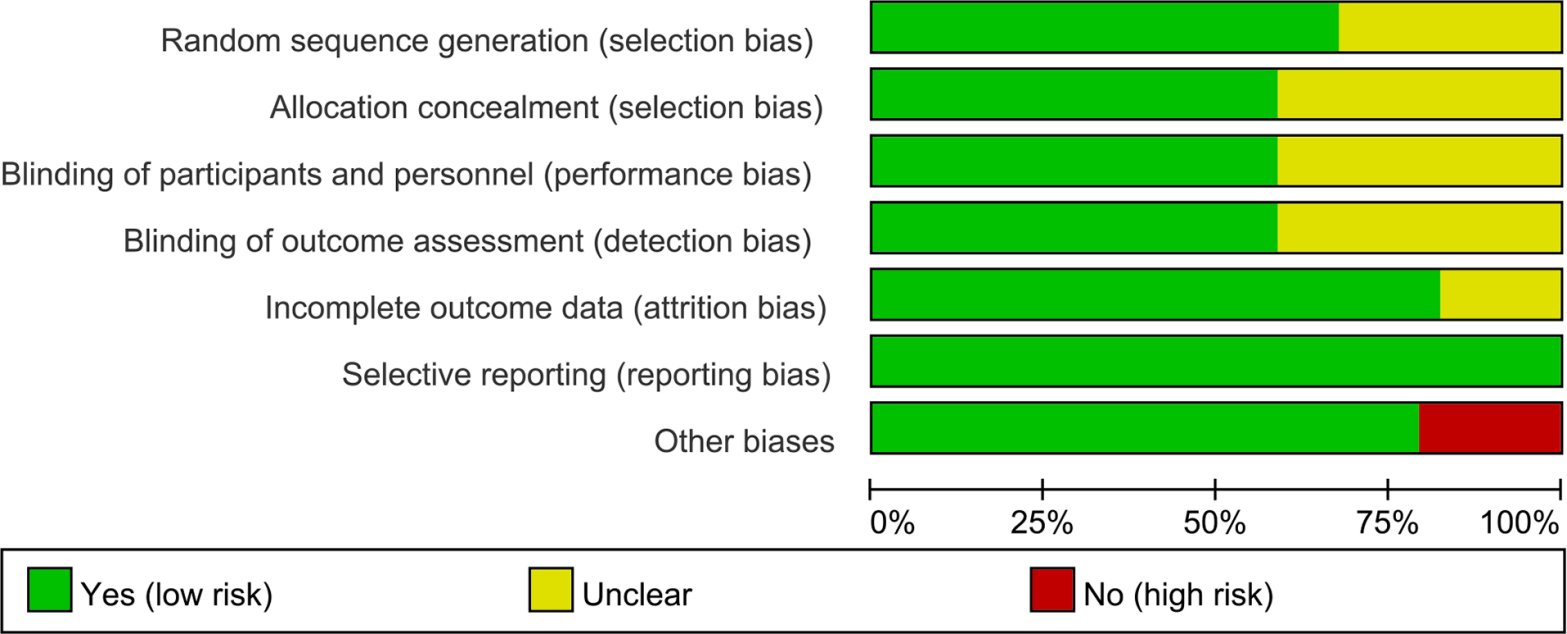
Risk of bias graph.

**Figure 3:**
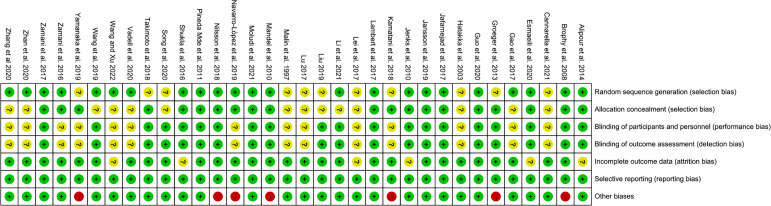
Risk of bias summary.

The summary and graph of risk of bias ware shown in **Figures 2**, **3**.

#### 3.3.1 Sequence generation and allocation concealment

Twenty-three (23) RCTs (32–34, 36–42, 44, 47–54, 56, 57, 60, 63, 64, 66, 68) describe the random sequences generating method and were rated as low risk of bias, while other RCTs did not described the generating methods, and were rated as unclear risk of bias.

Twenty (20) RCTs (32–34, 36–40, 44, 45, 47–53, 56, 57, 59, 61, 62, 66) used allocation concealment (using identical-looking drugs, or allocation methods that were imperceptible to patients and physicians) and were therefore assessed as low risk of bias. Other RCTs did not describe whether allocation concealment was performed, the information was unclear, and therefore were assessed as unclear risk of bias.

#### 3.3.2 Blinding

Twenty (20) RCTs (32–34, 36, 37, 39, 40, 45, 47–60, 66) described blinding of participants and personnel and blinding of outcome assessment, and were therefore assessed as low risk of bias. Other RCTs did not describe whether blinding was performed and were therefore assessed as unclear risk of bias.

#### 3.3.3 Incomplete outcome data and selective reporting

Alipour et al., 2014 (32–34), Esmaeili et al., 2020 (40), Jenks et al., 2010 (48), Wang and Xu 2022 (64), Lei et al., 2017 (65), Shukla et al., 2016 (66) had incomplete outcome data and did not report the use of intent-to-treat, and were therefore assessed as having an unclear risk of bias. The other RCTs do not have incomplete outcome data. All RCTs reported outcomes assessed in the protocol and were therefore assessed as low risk of bias.

#### 3.3.4 Other potential bias

Mandel et al., 2010 (36), Navarro-López et al., 2019 (44), Groeger et al., 2013 (45), Nilsson et al., 2018 (56, 57), Yamanaka et al., 2019 (61), Kamatani et al., 2018 (62) was funded by a pharmaceutical company and was therefore assessed to be at high risk of bias. Brophy et al., 2008 (49) conducted an Internet-based survey without face-to-face patient contact and were therefore assessed to be at high risk of bias. The others were rated as low risk of bias.

### 3.4 Probiotics for RA

Alipour et al., 2014 treated patients with *L. casei* 1*10^8 CFU and found improvements in CRP levels, tender and swollen joint counts, global health (GH) scores and DAS28 compared to placebo (P < 0.05). At the end of the study, more patients in the probiotic group had a moderate response to treatment (P < 0.05). Mandel et al., 2010 found a statistically significant improvement in joint pain and a reduction in CRP in patients receiving *Bacillus coagulans* GBI-30, 6086 2*10^8 CFU compared to placebo. Vadell et al., 2020 found no significant difference in DAS28-ESR compared with controls after administration of anti-inflammatory foods (including Lactobacillus plantarum 299v) (P = 0.116). Esmaeili et al., 2020 found that no significant differences in measured parameters were observed between the probiotic and placebo groups (P>0.05). Cannarella et al., 2021 found that probiotics improved white blood cell counts, TNF-α (P = 0.004) and IL-6 plasma levels (P < 0.05). However, no differences were observed in CRP, ESR, DAS28 between the two groups (P>0.05). Since data from the above RCTs could not be combined for meta-analysis, they are described only. The results of the meta-analysis are shown below.

#### 3.4.1 Efficacy indicators

**Figure 4:**
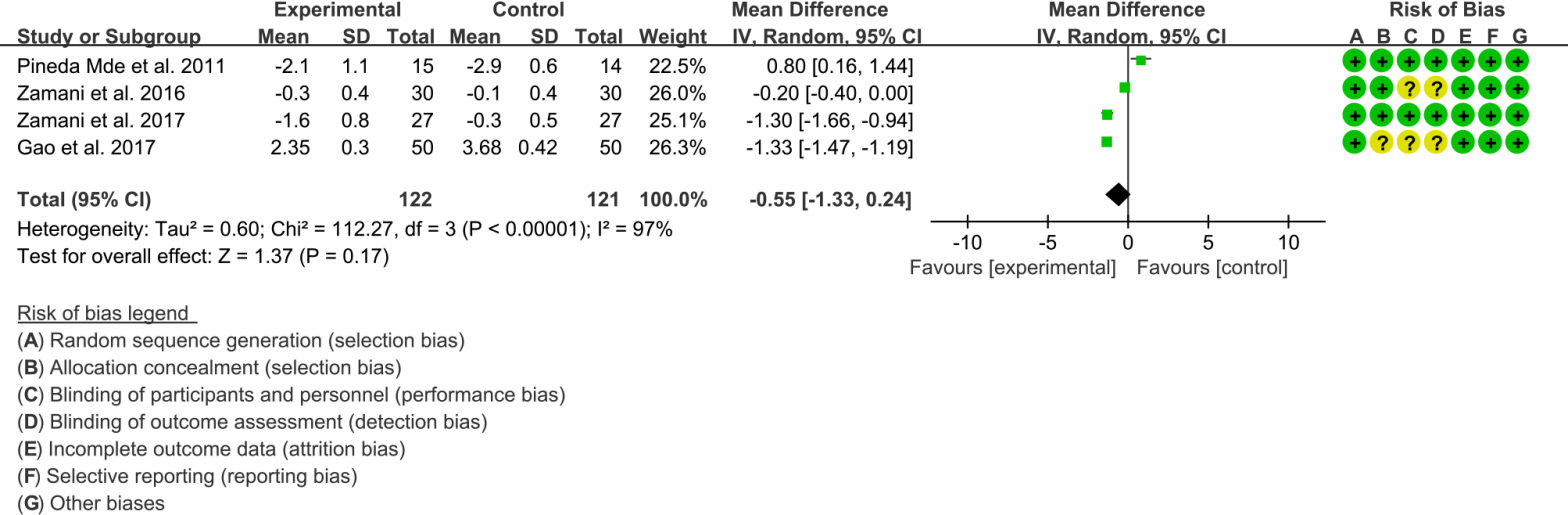
The results of DAS28 (CI, confidence interval; SD, standard deviation).

**Figure 5:**
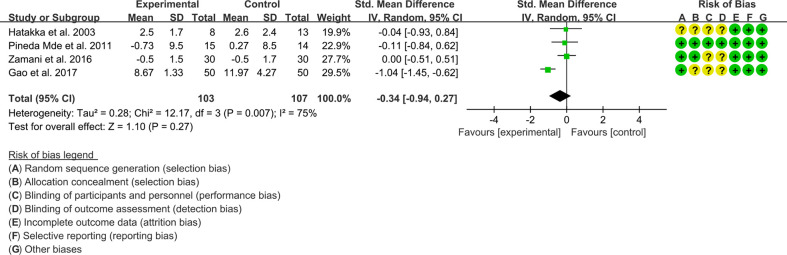
Tender joint count (CI, confidence interval; SD, standard deviation).

**Figure 6:**
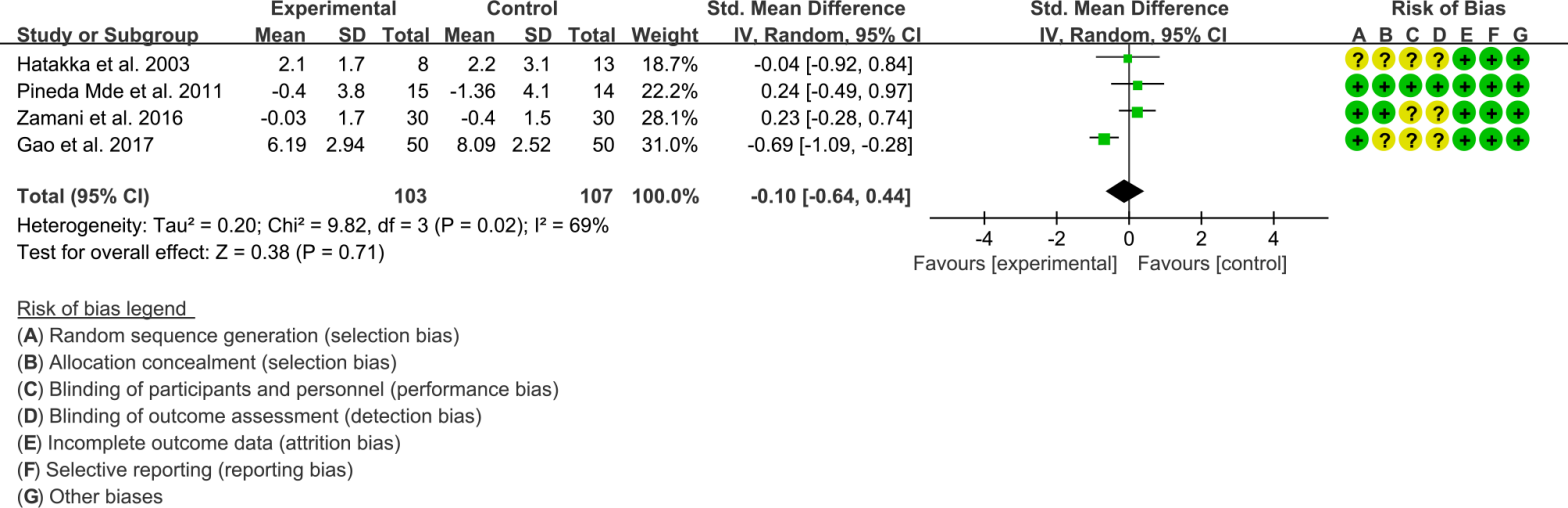
Swollen joint count (CI, confidence interval; SD, standard deviation).

(1) DAS28: Four (4) RCTs reported the DAS28 data that can be meta-analyzed. The result of heterogeneity analysis was I2 = 97% and P<0.00001, which showed that there was statistical heterogeneity among the 4 studies, so the random effects model was used. The results of Meta analysis showed that the difference of DAS28 between experiment group and control group was of no statistical significance [WMD -0.55 (-1.33, 0.24), P=0.17] (**Figure 4**).

(2) Tender joint count: Four (4) RCTs reported the tender joint count data that can be meta-analyzed. The result of heterogeneity analysis was I2 = 75% and P=0.007, which showed that there was statistical heterogeneity among the 4 studies, so the random effects model was used. The results of Meta analysis showed that the difference between the experimental group and control group is of no statistical significance [SMD -0.34 (-0.94, 0.27), P=0.27] (**Figure 5**).

(3) Swollen joint count: Four (4) RCTs reported the swollen joint count data that can be meta-analyzed. The result of heterogeneity analysis was I2 = 69% and P=0.02, which showed that there was statistical heterogeneity among the 4 studies, so the random effects model was used. The results of Meta analysis showed that the difference between the experimental group and control group is of no statistical significance [SMD -0.10 (-0.64, 0.44), P=0.71] (**Figure 6**).

#### 3.4.2 Inflammatory indicator

**Figure 7:**
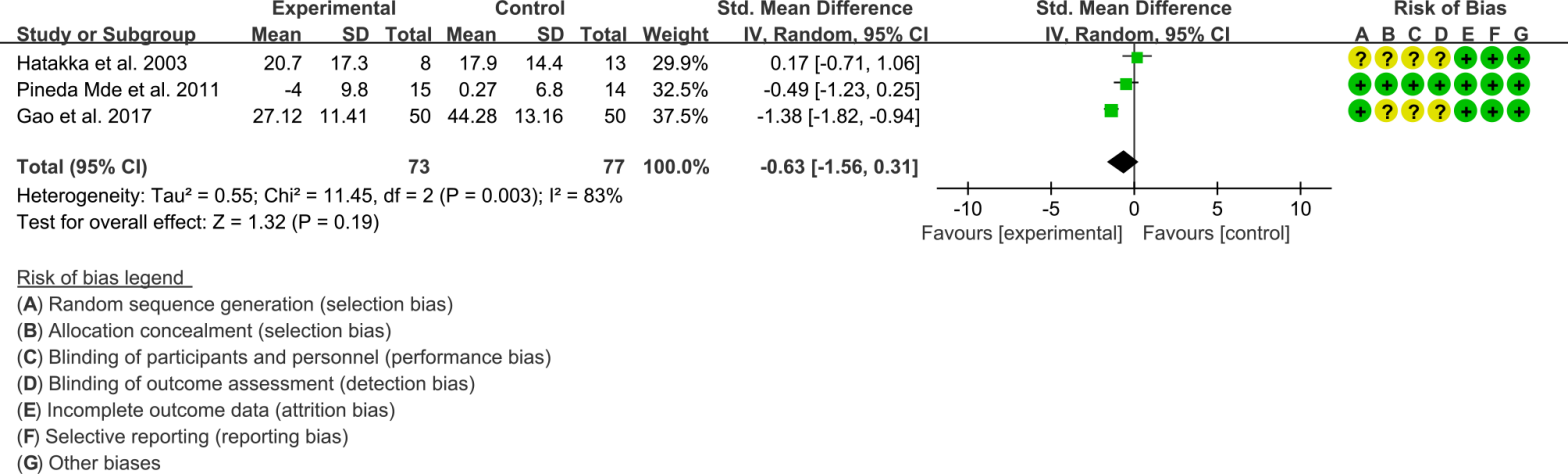
The results of ESR (CI, confidence interval; SD, standard deviation).

(1) ESR: Three (3) RCTs reported the ESR data that can be meta-analyzed. The result of heterogeneity analysis was I2 = 83% and P=0.003, which showed that there was statistical heterogeneity among the 3 studies, so the random effects model was used. The results of Meta analysis showed that the difference between the experimental group and control group is of no statistical significance [SMD -0.63 (-1.56, 0.31), P=0.19] (**Figure 7**).

**Figure 8:**
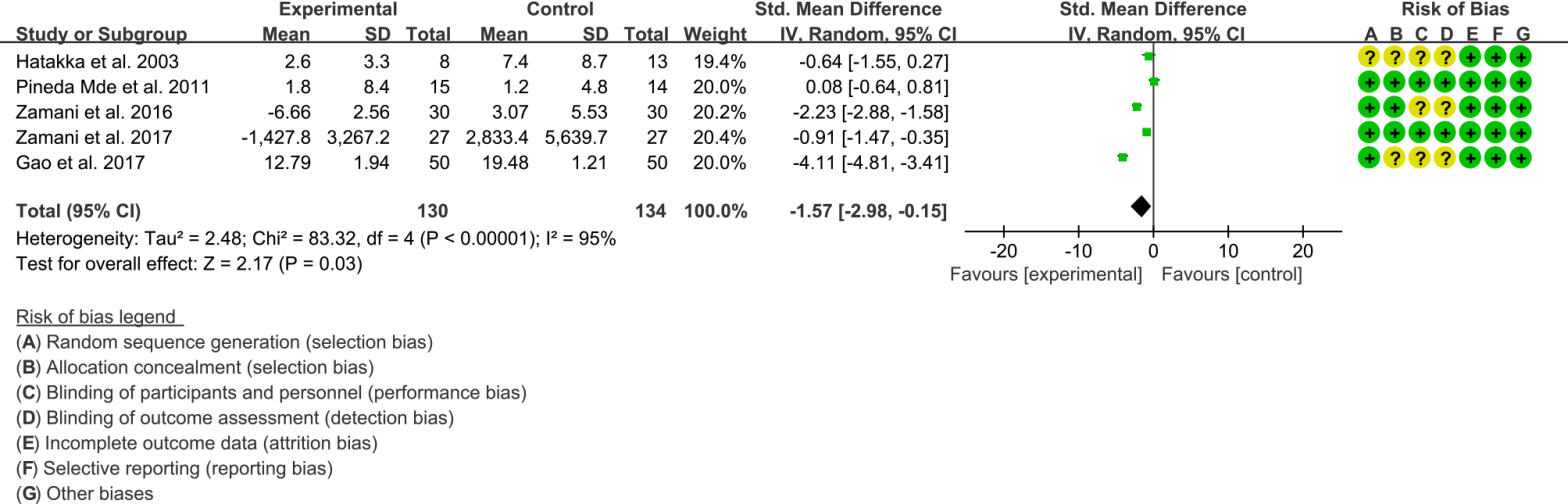
The results of CRP (CI, confidence interval; SD, standard deviation).

**(**2) CRP: Five (5) RCTs reported the CRP data that can be meta-analyzed. Since The result of heterogeneity analysis was I2 = 95% and P<0.00001, which showed that there was statistical heterogeneity among the 5 studies, so the random effects model was used. The results of Meta analysis showed that there was a statistical difference between the experimental group and the control group (P=0.03), which indicates that curcumin may decrease CRP [SMD -1.57 (-2.98, -0.15)] (**Figure 8**).

#### 3.4.3 Adverse events

Five RCTs (32–34, 36, 37, 41, 42) reported adverse events. No adverse events were observed in Alipour et al., 2014 (32–34), Mandel et al., 2010 (36), Pineda Mde et al., 2011 (37). Vadell et al., 2020 (41) observed 13 gastrointestinal adverse events in the intervention group and 4 gastrointestinal adverse events in the control group, mainly stomach pain, gas, diarrhea, heartburn and nausea. Gao et al., 2017 (42) found that 1 patient felt mild pain and discomfort in the lower abdomen after 3 days of oral treatment, and 1 patient had increased stool frequency; no serious adverse events were observed in any of the patients. In addition, five RCTs (35, 38–40, 43) did not report whether adverse events were observed, possibly because they did not monitor for adverse events, or did not observe adverse events indeed.

### 3.5 Probiotics for psoriasis

#### 3.5.1 PASI score

**Figure 9:**
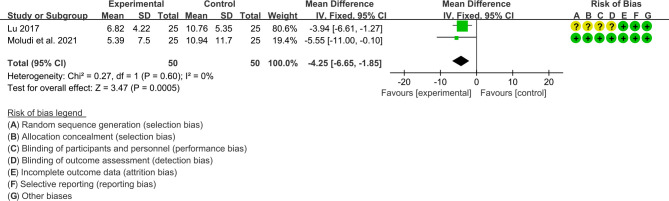
The results of PASI (CI, confidence interval; SD, standard deviation).

Three RCTs reported PASI score. The results of Navarro-López et al., 2019 showed that the improvement of PASI in the probiotic group was better than that in the placebo group (P>0.05); however, because it did not provide specific values, it could not be integrated into the meta-analysis. The result of heterogeneity analysis was I2 = 0% and P=0.60, which showed that the heterogeneity among the 2 studies was low, so the fixed effects model was used. The summary results showed that the PASI score in probiotic group was lower than the control group (WMD -4.25 [-6.65, -1.85], P=0.0005; fixed effect model) (**Figure 9**).

#### 3.5.2 CRP, TNF-α and IL-6

Two RCTs reported CRP and IL-6 levels. Both Groeger et al., 2013 and Moludi et al., 2021 found that CRP was reduced after the intervention of probiotics (P<0.05). However, for IL-6, Moludi et al., 2021 found that IL-16 was lower after the intervention of probiotics (P<0.05), while Groeger et al., 2013 showed that there was no significant difference between the probiotics intervention and the placebo group (P>0.05). Groeger et al., 2013 also reported TNF-α level and showed it was lower after the intervention of probiotics (P<0.05).

#### 3.5.3 Adverse events

Two RCTs reported adverse events. Navarro-López et al., 2019 (44) showed a low incidence of adverse events, and no serious adverse events occurred in both groups. Moludi et al., 2021 (47) showed that 12% of patients in the placebo group and 8% of the patients in the probiotic group experienced gastrointestinal reactions, suggesting that patients tolerated probiotics well. In addition, two RCTs (45, 46) did not report whether adverse events were observed, possibly because they did not monitor for adverse events, or did not observe adverse events indeed.

### 3.6 Probiotics for spondyloarthritis

Only two RCTs reported the results of probiotics for Spondyloarthritis. Jenks et al., 2010 found no significant difference in mean BASFI and BASDAI after probiotic intervention compared with placebo (P>0.05). It also found adverse events in 14 patients in the probiotic group (12 in the placebo group), with no statistically significant difference, including include changes in bowel habits, nausea, vomiting, abdominal pain, and increased breathing. Brophy et al., 2008 used the Internet to recruit patients, send drugs to patients by post, and finally obtain patient feedback through the Internet. It found no significant differences in global health status, severity of intestinal symptoms or arthritis, and incidence of adverse events between the probiotic and control groups (P>0.05).

### 3.7 Probiotics for hyperuricemia and gout

#### 3.7.1 Serum uric acid

**Figure 10:**
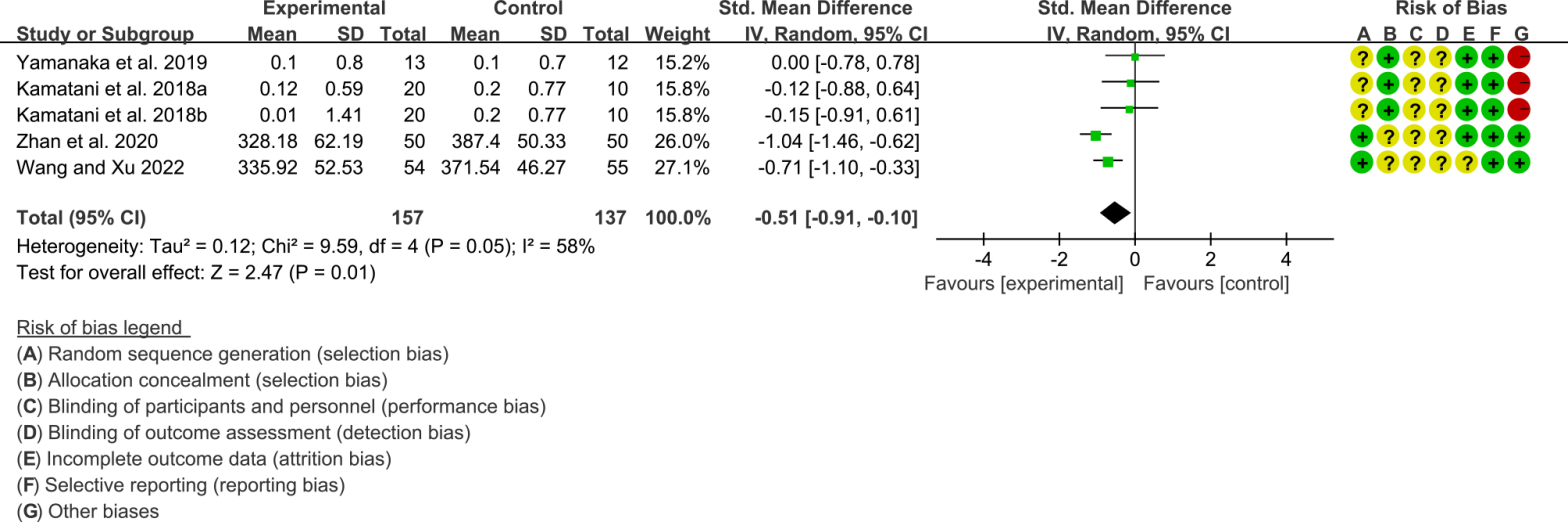
Serum Uric acid (CI, confidence interval; SD, standard deviation).

Four RCTs (157 participants in experimental group and 137 participants in control group) reported uric acid. The heterogeneity test showed P=0.05, I2 = 58%, indicating high heterogeneity, and the random effects model was used for analysis. The summary result showed that uric acid in the probiotic group was lower than that in the control group (SMD -0.51 [-0.91, -0.10], P=0.01; random effect model) (**Figure 10**).

#### 3.7.2 Adverse events

None of the four RCTs reported relevant adverse events, possibly because they were not observed or were not monitored. It is expected that future studies will report in detail the adverse events of probiotics intervention in hyperuricemia or gout.

### 3.8 Probiotics for osteoporosis and osteopenia

#### 3.8.1 BMD

**Figure 11:**
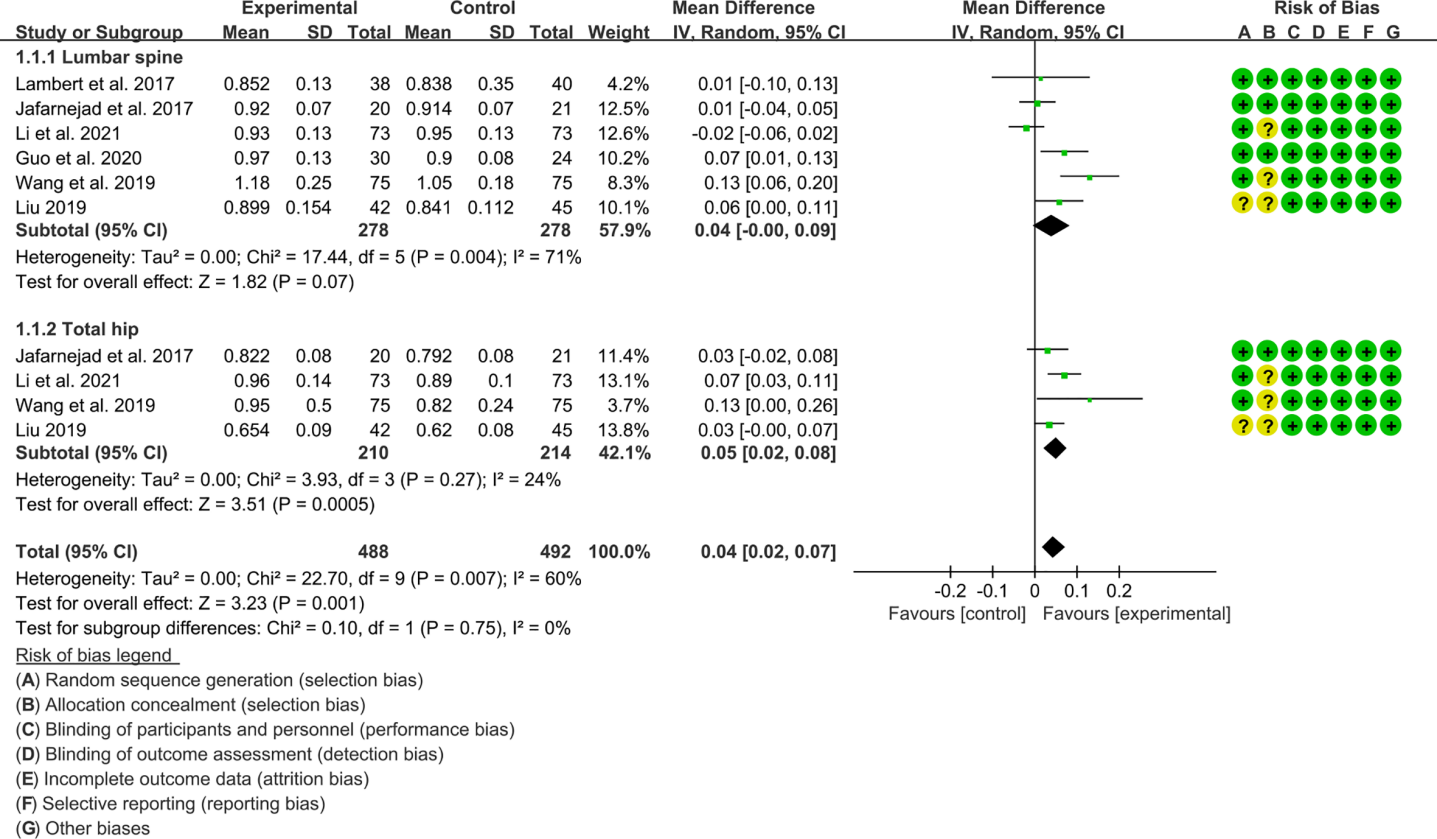
The absolute value of BMD (CI, confidence interval; SD, standard deviation).

Seven (7) RCTs reported the absolute value of BMD, and 3 RCTs reported the percentage of BMD improvement. For the absolute value of BMD, the result of heterogeneity analysis showed that the heterogeneity was high in lumbar spine’s BMD (I2 = 71% and P=0.004) but low in total hip’s BMD (I2 = 24% and P=0.27), the random effects model was used. In lumbar spine subgroup, the difference between experimental group and control group was of no statistical significance (WMD 0.04 [-0.00, 0.09], P=0.07; random effect model). In total hip subgroup, the improvement of BMD in experimental group was higher (WMD 0.05 [0.02, 0.08], P=0.0005; random effect model) (**Figure 3**). The summary results also showed that the improvement of BMD in the experimental group was higher (WMD 0.04 [0.02, 0.07], P=0.001, random effect model) (**Figure 11**).

**Figure 12:**
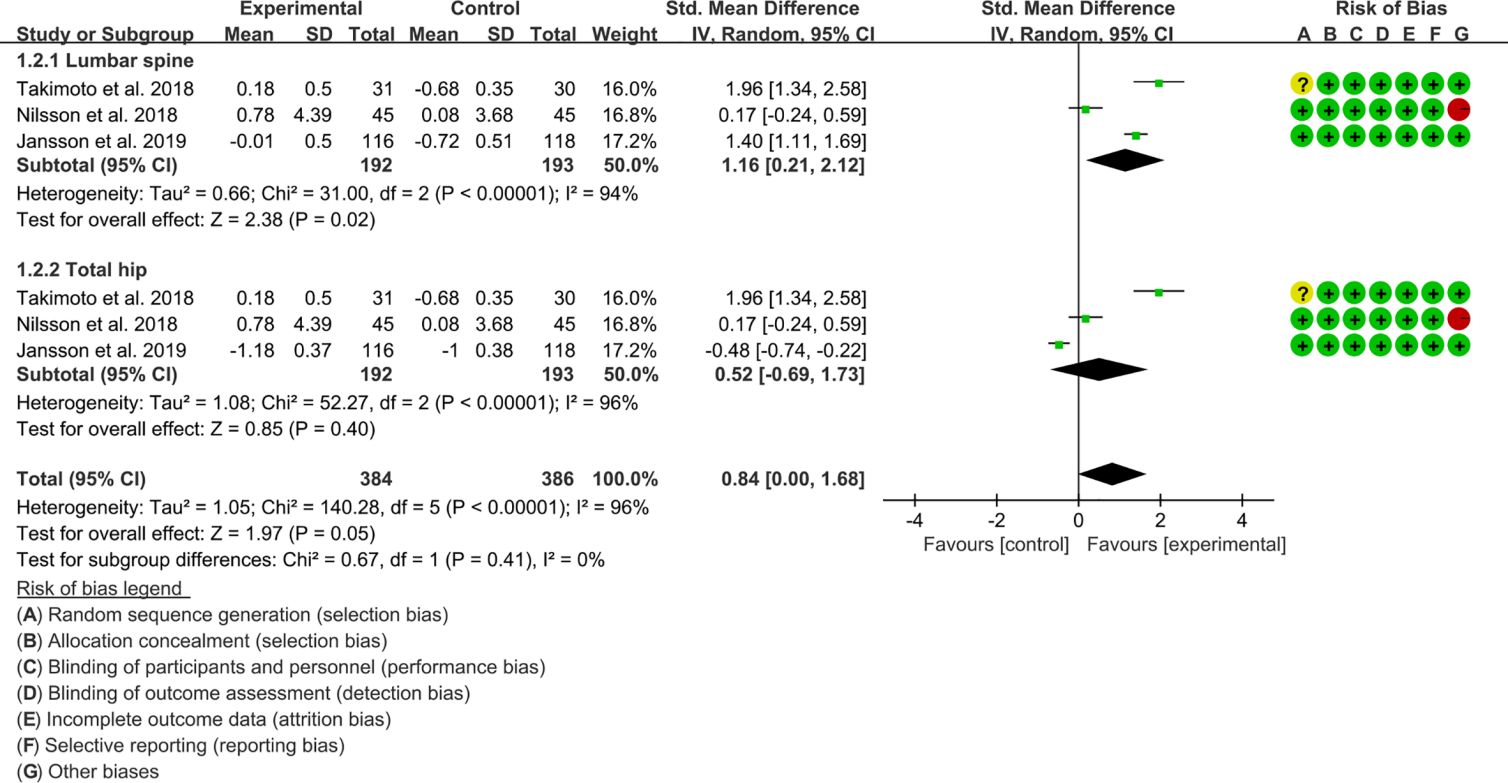
The percentage of BMD improvement (CI, confidence interval; SD, standard deviation).

For the percentage of BMD improvement, the result of heterogeneity analysis showed that the heterogeneity was high in lumbar spine’s BMD (I2 = 94% and P<0.00001) and total hip’s BMD (I2 = 96% and P<0.00001), the random effects model was used. In lumbar spine subgroup, the improvement of BMD in experimental group was higher (SMD 1.16 [0.21, 2.12], P=0.02; random effect model) (**Figure 3**). In total hip subgroup, the difference between experimental group and control group was of no statistical significance (SMD 0.52 [-0.69, 1.73], P=0.40; random effect model). The summary results also showed that the difference of BMD between the two groups was of no statistical significance (SMD 0.84 [0.00, 1.68], P=0.05, random effect model) (**Figure 12**).

#### 3.8.2 Adverse events

Five (5) RCTs reported the adverse events (52–54, 56, 57, 59). Lambert et al., 2017 (53) reported gastrointestinal adverse events in 2 patients in the probiotic group and 1 patient in the control group. Takimoto et al., 2018 (59) showed that no adverse events were observed. Nilsson et al., 2018 (56, 57) showed that there were 36 adverse events in the probiotic group and 39 in the control group, and there was no statistical difference between the two groups (P>0.05). Jansson et al., 2019 (52) showed that there were 30 adverse events in the probiotic group and 32 in the control group, and there was no statistical difference between the two (P>0.05), most of which were gastrointestinal reactions. Li et al., 2021 (54) showed that there were 5 cases of adverse events in the probiotic group and 7 cases in the control group, mainly nausea, vomiting, diarrhea and dizziness, and the difference was not statistically significant (P>0.05). In addition, five RCTs (50, 51, 55, 58, 60) did not report whether adverse events were observed, possibly because they did not monitor for adverse events, or did not observe adverse events indeed.

### 3.9 Probiotics for OA

Only one RCT reported probiotics for OA. Lei et al., 2017 treated 215 patients with *L. casei* Shirota and another 218 with placebo. They found that after 6 months of treatment, compared with the placebo group, patients in the probiotic group had significantly improved WOMAC and VAS scores, and decreased serum hs-CRP levels (P<0.05). They also claim that no serious adverse events were observed throughout the study.

### 3.10 Probiotics for JIA

Only two (2) RCTs reported probiotics for JIA. Shukla et al., 2016 found that the improvement of mJSpADA was not statistically different between the probiotic group and the control group (P>0.05). IL-10 levels were decreased in the probiotic group compared with the placebo group (P < 0.01). It also reported that there was no significant difference in adverse reactions between the probiotic group and the placebo group (11 vs 9, P>0.05), mostly diarrhea, abdominal pain, mild infection and flatulence. Malin et al., 1997 randomly assigned 30 adolescent patients to receive either *L. rhamnosus* GG or bovine colostrum or a lyophilized powder of bovine immunized colostrum for two weeks. They found that the probiotic group increased the number of immune cells secreting IgA and IgM (P<0.05). Fecal urease activity associated with mucosal tissue damage was decreased in the probiotic group, while increased in the control group (P<0.05). They suggest that oral administration of *L. rhamnosus* GG has the potential to strengthen the mucosal barrier mechanism of juvenile chronic arthritis. In addition, those two RCTs (66, 67) did not report whether adverse events were observed, possibly because they did not monitor for adverse events, or did not observe adverse events indeed.

### 3.11 Probiotics for inflammatory bowel disease arthritis

Only Zhang et al., 2020 (68) reported probiotics for inflammatory bowel disease arthritis. They treated 120 patients with probiotic therapy, narrative medical education therapy, probiotics combined with narrative medical education therapy, and primary care therapy. They found that the patients in the probiotics combined with narrative medical education group had the longest total sleep time, the shortest sleep latency, and the highest sleep efficiency, suggesting that this therapy could help improve the sleep quality of patients. In addition, Zhang et al., 2020 (68) did not report whether adverse events were observed, possibly because they did not monitor for adverse events, or did not observe adverse events indeed.

## 4 Discussion

**Figure 13:**
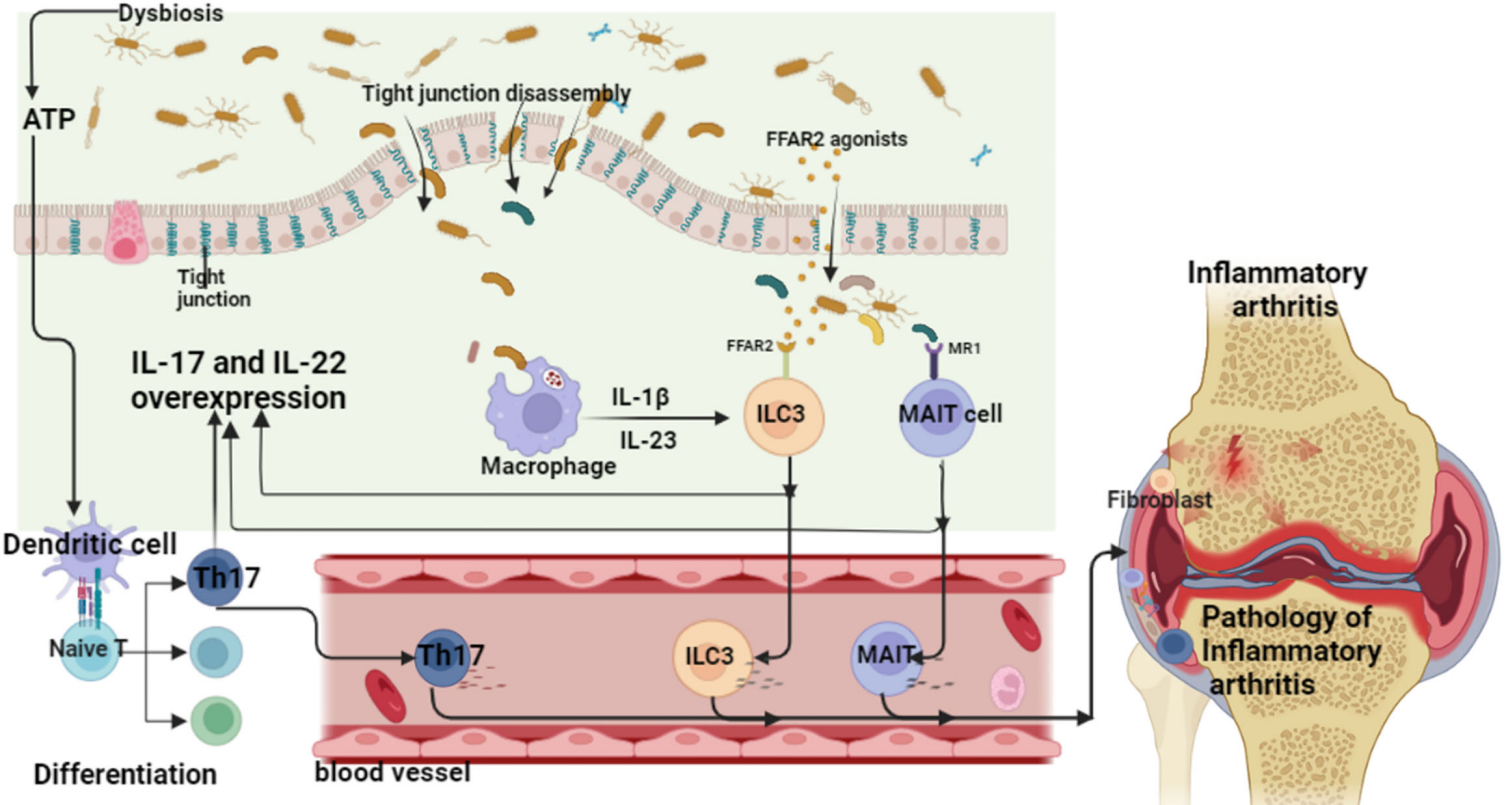
Inflammatory arthritis - the pathogenic mechanism of the “gut-arthritis” axis (ATP, adenosine triphosphate; IL, interleukin; ILC, innate lymphoid cells; MAIT cell, mucosal associated invariant T; Th17, T helper cell 17).

There is increasing evidence that non-human genetic factors, especially the human microbiota, may contribute to the development of inflammatory arthritis (e.g., RA and spondyloarthritis) in genetically susceptible individuals (72–74). There has been an increasing number of studies investigating the gut community as a determinant of the pathogenesis of inflammatory arthritis. Evidence from multiple epidemiological and translational studies suggests that interactions between mucosal sites and dysregulated microbiota may contribute to the development of inflammatory arthritis (75–78). Studies have shown that in most inflammatory arthritis in the preclinical stage of arthritis, there is already a change in the microbial microbiota of the individual patient. This suggests that gut dysbiosis plays an important role in the development of inflammatory arthritis and systemic inflammation throughout the chronic course (79–86). Therefore, the current hypothesis model is mainly that the inflammatory “gut-arthritis axis” is the pathogenic pathway of inflammatory arthritis. The gut is composed of the most innate and adaptive immune cells in the human body, so it is generally considered to be the largest immune organ in the human body (87). The possible complex interplay between gut microbiota disturbances and genetic factors (the immune system susceptible to autoimmunity) may provide the basis for the development of pathological processes such as systemic inflammation in inflammatory arthritis (88–90). Basic and clinical studies suggest that these alterations in gut microbiota disturbances may occur before disease onset, and to some extent represent hidden triggers of systemic inflammation (24, 91). After initiation of the initiating factors, the links between barrier dysfunction, intestinal inflammation and arthritis reciprocate to form the “gut-arthritis” axis. Among them, dysbiosis may lead to subclinical intestinal inflammation in patients and promote the abnormal activation of specific innate and adaptive immune response pathways. Mucosal lesions due to inflammation exhibit reduced intestinal barrier function, and abnormally activated immune cells are also transported from intestinal sites to secondary lymphoid organs and recirculation of arthritic joints (92, 93) (**Figure 13**). Therefore, therapies targeting the entero-arthritis axis have emerged as new therapeutic measures for inflammatory arthritis.

At present, probiotics are live microorganisms that are beneficial to the human body and are of great significance to maintain the intestinal microecological balance. Several clinical studies have shown that probiotics and their metabolites or probiotic fermented foods have received great attention in improving inflammatory arthritis. Among them, *Bifidobacterium* (such as *B. longum*, *B. breve*, *B.infantis*) and *Lactobacillus* (such as *L. helveticus*, *L. rhamnosus*, *L. plantarum* and *L.casei*) are the most widely used probiotics (94). According to the WHO, probiotics, as active microorganisms, can have beneficial effects on the body if taken in appropriate doses (75). Probiotics can restore the balance of microbiota in the gut in various ways, including regulating gut immunity or competing with other gut microbes for nutrients, resulting in a competitive inhibitory effect (76). More and more researchers have found that probiotics can regulate the immune system of humans or animals (77, 78). A systematic review and meta-analysis of chronic intestinal inflammatory diseases showed that in 8 case-control studies and 1 randomized controlled study, more than 45% of patients achieved clinical remission after probiotic intervention (79). Some researchers have also discovered the therapeutic effect of probiotics on inflammatory arthritis. Therefore, we conducted a comprehensive and extensive systematic review and meta-analysis on the efficacy and safety of probiotics on the mechanism of inflammatory arthritis.

### 4.1 Probiotics for RA

RA is a chronic systemic autoimmune disease characterized by joint lesions, in which autoantibodies are mainly represented by rheumatoid factor and anti-cyclic citrullinated peptide antibody (ACPA) (95). It is characterized by joint swelling and pain, followed by deformity and disability (96), and is accompanied by damage to internal organs, such as lungs, heart, and kidneys. Epidemiological surveys show that the prevalence of RA is 0.3% and 1%, and it is one of the most common autoimmune diseases (97). Current research shows that RA is caused by the combined action of genetic factors and environmental factors. Among environmental factors, immune abnormalities caused by immune cell imbalance have been confirmed to be involved in the occurrence and development of RA (98). These include T, B lymphocytes, macrophages, neutrophils, etc., and imbalances in the proportion of Th1, Th2 and Th17 cells and immune damage caused by cytokines IL-1, IL-2, IL-17, IFN-γ and TNF-α (99). It is a key factor leading to chronic inflammation in RA. In particular, adaptive immunity dominated by CD4+ T cells plays an important role in initiating and maintaining the characteristics of the autoimmune response in rheumatoid arthritis. Among them, Th1 and Th17 cells in CD4+ T cells are important drivers of RA, which activate macrophages and recruit other inflammatory cells to inhibit Treg-mediated immune tolerance (100).

The current study showed significant differences in the fecal microbiota of RA patients compared with healthy subjects. The levels of *Bifidobacterium*, *Bacteroides*, *Lactobacillus* and other probiotics in the feces of RA patients were significantly decreased, while the levels of *Escherichia coli* and Enterococcus were significantly increased (38, 101, 102). These gut microbiota are involved in the innate immune response pathway and the immune abnormalities of the acquired immune response pathway in the pathogenesis of RA (103). The study found that gavage of Lactobacillus could correct the imbalance of intestinal bacteria in mice with collagen-induced arthritis (CIA), and at the same time reduce the expression of cytokines IL-12, IFN-γ, TGF-β and IL-6 that induce Th1 and Th17 cell differentiation. That is, *Lactobacillus* can alleviate mouse RA by regulating CD4+ T subset-related cytokines (104). Another study (105) also found that the B cells and Tfh cells associated with antibody production in the inguinal lymph nodes were significantly reduced in CIA mice fed *L. helveticus* SBT2171. In terms of Treg cells regulating immunity, the alleviating effect of probiotics on CIA rats was related to the time of intervention. *Bifidobacterium* preventive intervention is more likely to improve the intestinal microecology, increase the concentration of short-chain fatty acids, increase the frequency of Treg cells, and relieve the symptoms of CIA rats than the therapeutic intervention (106). RA symptoms are closely related to the overproduction of pro-inflammatory factors and the activation of intracellular pro-inflammatory signals. In addition, after different doses of compound probiotics (*B. breve, L. casei, L. bulgaricus, L. rhamnosus* and *L. acidophilus*) intervened in CIA mice, the degree of joint swelling and pain sensitivity of mice were reduced, and the infiltration of inflammatory cells was also reduced. Serum IL-1β levels decreased, and the activated p38 mitogen-activated protein kinase (MAPK) inflammatory pathway in the spinal cord was inhibited (107). In addition, the reduction of inflammatory factors by gut microbes by regulating redox balance may be one of the mechanisms by which probiotics alleviate rheumatoid arthritis (108). Gavage og *L. casei* in CIA rats can significantly increase the types and abundance of gut microbes. They found that enterobacterial abundance was inversely correlated with pentose phosphate pathway activating enzyme activity. This indicates that *L. casei* can inhibit the activation of the pentose phosphate pathway, maintain redox balance, and reduce the production of pro-inflammatory factors IL-1β, IL-6, and IL-17, thereby alleviating rheumatoid arthritis in rats (109).

In this meta-analysis, although only CRP in this meta-analysis showed lower in the probiotic group, results from some studies showed improvement in RA. Alipour et al., 2014 treated patients with *L. casei* 1*10^8 CFU and found improvements in CRP levels, tender and swollen joint counts, GH scores and DAS28 compared to placebo; meanwhile, more patients in the probiotic group had a moderate response to treatment. Mandel et al., 2010 found a statistically significant improvement in joint pain and a reduction in CRP in patients receiving *Bacillus coagulans* GBI-30, 6086 2*10^8 CFU compared to placebo. Cannarella et al., 2021 found that probiotics improved white blood cell counts, TNF-α (P = 0.004) and IL-6 plasma levels. This suggests that *L. casei*, *Bacillus coagulans*, *L. acidophilus, Lactococcus lactis, B. lactis* and *B. bifidum* may have potential curative effects on RA, and more research on the intervention of these probiotics in RA can be carried out in the future. However, since some of these RCTs do not report the details of random sequence generation methods, allocation concealment methods, and blinding methods, the quality was degraded. Hence, the results need to be interpreted with caution. In addition, some RCTs did not report adverse events, so more RCTs reporting efficacy and safety are needed to further explore the treatment of RA with probiotics.

### 4.2 Probiotics for psoriasis

Psoriasis affects about 2% of the global population and affects all age groups (110). Psoriasis may be related to genetic factors, immune dysfunction, and environmental factors. As a chronic inflammatory disease, psoriasis can be caused by a variety of factors, and the disease is prone to relapse over time. Studies have shown that the pathogenesis of psoriasis is mainly related to the helper T cell (Th cell) 17/IL-23 axis. The intestinal microbiota can participate in the differentiation of T cells, such as segmented filamentous bacteria can induce pro-inflammatory responses in Th17 cells in the intestine (111). Experiments have shown that both short-chain fatty acids (SCFAs)-producing microbiota and SCFAs can act as potent regulators of T cells in the context of T cell-mediated inflammation (112–115). Among them, *Clostridium* is the main producer of SCFAs, which can induce the production of IL-10 in the colon, and at the same time increase the number of regulatory T cells (Treg cells) in the mucosa, playing a key role in intestinal homeostasis (113). In terms of microbiota distribution, there are certain differences between healthy people and patients with psoriasis. For example, the abundance of *Akkermansiamuciniphila* is decreased in patients with psoriasis (116). Experiments have shown that increasing *A. muciniphila* can reduce obesity and repair the damaged intestinal barrier in mice caused by diet (117). Therefore, the decreased abundance of *A. muciniphila* in the gut of patients with psoriasis can lead to impairment of the intestinal barrier, which in turn induces the development of psoriasis. The gut microbiota of patients with psoriatic arthritis was the same as that of patients with cutaneous psoriasis, and both beneficial bacteria were reduced. It is manifested as Th1 and Th17 cell-driven inflammatory arthritis with new bone formation, suggesting that spondyloarthritis and psoriasis may share a common pathogenic pathway (118). The study found disturbances in the gut microbiota in both people with spondyloarthritis and rodent models. The results of ileal biopsy showed that the *Porphyromonas*, *Lachnospiraceae*, *Verrucobacterium*, *Rikenbacteriaceae* and *Bacteroidetes* are rich (119). Meanwhile, transplantation of *Escherichia coli* from Crohn’s disease patients with spondyloarthritis into germ-free mice induces a Th17 cell response. It also increases the severity of colitis or arthritis in disease-sensitive IL-10-deficient or potassium/BxN mice (120). In summary, the intestinal microbiota is involved in the occurrence and development of psoriasis and psoriatic arthritis. Deng et al. (121) found that reducing *B. breve* CCFM1078 and *B. adolescentis* CCFM667 and *L. paracasei* CCFM1074 and *L. reuteri* could reduce the levels of IL-23/Th17 axis inflammatory factors in animal models of psoriasis. Its specific action pathway is to promote the production of short-chain fatty acid SCFAs and then reduce the level of IL-23/Th17 axis inflammatory factors, thereby alleviating psoriasis. This meta-analysis also showed that the PASI score of patients with psoriasis decreased after probiotic intervention. However, due to the small number of RCTs, meta-analysis of outcomes such as inflammatory factors could not be performed and the results need to be interpreted with caution. In addition, some RCTs did not report adverse events, hence, more RCTs reporting efficacy and safety are needed to further explore the treatment of psoriasis with probiotics.

### 4.3 Probiotics for spondyloarthritis

Ankylosing spondylitis (AS) is a chronic progressive rheumatic disease that is mainly characterized by chronic inflammation of the axial joint and can involve internal organs and other tissues. The incidence of AS ranges from 0.1% to 1.6% (122), the age of first onset is 20 to 30 years old, and it is more common in young men (123). AS is a common, highly hereditary immune arthropathy that occurs in genetically susceptible individuals exposed to unknown but possibly prevalent environmental triggers. Animal model studies have shown that germ-free SKG mice cannot develop the disease, but chlamydia infection can lead to axial and peripheral arthritis, psoriasis and uveitis, indicating that the intestinal microbiota plays an important role in the pathogenesis of AS (124). The study found that the intestinal microbiota structure of AS patients was significantly changed compared with the normal population. Compared with healthy controls, the ileum terminal ileum had higher abundances of five bacterial families: *Lachnospira, Prevotella, Rikenbacteriaceae, Porphyromonas*, and *Bacteroidetes* (125). Another study found that *Bacteroidetes, Firmicutes, Proteobacteria*, and *Actinobacteria* were the four major microbiota in the gut microbiota of AS patients and healthy controls. However, the abundance of actinomycetes in AS patients was significantly higher than that in the control group, especially Bifidobacterium, while the abundance of *Clostridium* and *Verrucobacterium* was lower, and Gram-negative *Enterobacteriaceae* and *Citrobacter* were relatively less (126). At present, the mechanism of action of gut microbiota in the etiology of AS is not fully understood. It is generally believed that intestinal microbiota may lead to the occurrence and development of AS through molecular simulation of HLA-B27, modification of autoantigens, destruction of tight junction proteins, increase of intestinal permeability and mediating abnormal immune responses of intestinal-associated lymphoid tissues (127). Asquith et al. believed that HLA alleles increased the risk of AS by interacting with the gut microbiome, and suggested that therapies targeting the microbiome may be effective in preventing or treating AS (128). Amdekar et al. have shown that *L. casei* may prevent arthritis by reducing prostaglandin levels and reducing joint inflammation in rats with collagen-induced arthritis (129). However, in this systematic review and meta-analysis, only two RCTs were included and showed no clinical effect, which indicates that the therapy (*Streptococcus salivarius* K12 1.6×10^8 CFU + *B. lactis* LAFTI B94 6.4×10^8 CFU + *L. acidophilus* LAFTI L10 6.4×10^8 CFU) in Jenks et al., 2010 (48) and the therapy (*L. salivarius* CUL61 6.25×10^9 CFU + *L. paracasei* CUL08 1.25×10^9 CFU + *B. infantis* CUL34 1.25×10^9 CFU + *B. bifidum* CUL20 1.25×10^9 CFU) in Brophy et al., 2008 (49) may not be effective in AS and potentially other probiotics could be considered for investigation. However, since Brophy et al., 2008 (49) conducted an Internet survey without face-to-face patient contact; and probiotics for spondyloarthritis involved only 2 RCTs, the reliability of the results needs to be interpreted with caution. In addition, some RCTs did not report adverse events, hence, more RCTs reporting efficacy and safety are needed to further explore the treatment of spondyloarthritis with probiotics.

### 4.4 Probiotics for hyperuricemia and gout

Gout is an inflammatory disease caused by purine metabolism disorder, decreased uric acid excretion or excessive production, resulting in elevated blood uric acid levels and deposition of uric acid crystals in joints and other connective tissues (130). The typical clinical manifestations of joint swelling and pain are not only joint deformities, but also kidney damage, cardiovascular and cerebrovascular diseases, etc., which seriously affect the quality of life of patients (131). The occurrence and development of gout is directly related to the level of blood uric acid in the body, and uric acid is the final metabolite of purine compounds. Therefore, regulating purine metabolism is very important for the pathogenesis and treatment of gout. Studies have found that there are two types of purines in the body, exogenous and endogenous. Reasonable adjustment of dietary structure to reduce the intake of exogenous purines is an effective method for clinically controlling acute gout attacks (132). As an important organ for digestion and absorption of food, the main function of the intestine is that the intestinal microbiota decomposes food into monosaccharides, which are then absorbed into the blood by small intestinal epithelial cells. If the intestinal microenvironment is disturbed, the intestinal microbiota will directly migrate to the extra-intestinal tissues, produce small molecules and participate in the blood circulation, and ultimately interfere with the intestinal Toll-like receptors and lead to the occurrence of rheumatic immune diseases (133, 134). Therefore, intestinal microbiota is closely related to the occurrence and development of gout by participating in the synthesis of purine metabolizing enzymes and the release of inflammatory factors. Studies have confirmed that compared with healthy people, the abundance of intestinal microbiota in gout patients is significantly lower (135). At present, many studies have found that the intestinal microbiota in gout patients is significantly different from that of healthy people. Ren et al. found that there is an imbalance of intestinal microbiota in patients with hyperuricemia, which is mainly manifested as a decrease in the number of physiological microbiota (136). However, the number of *Enterobacteriaceae* and total anaerobic bacteria increased significantly, indicating that the increase in serum uric acid may be related to the reduction of probiotics such as *Bifidobacterium* and *Lactobacillus* (136). Guo et al. found that the number of *Bacteroides caccae* and *Bacteroides xylanisolvens* in gout patients was significantly increased, while *Faecalibacterium prausnitzii* and *Bifidobacterium pseudocatenulatum* were relatively absent (137). Shao et al. (138) found that the diversity of gut microbiota in most gout patients showed a downward trend, with changes in opportunistic pathogens such as anaerobic bacteria and *Bacteroidetes*. In addition, Xing et al. (139) found that the number of *Clostridium* spp. in gout patients was significantly lower than that in healthy people. Some researchers have also used 1 H-NMR and Illumina Miseq technology to study the metabolic profile and microbial community of fecal extracts from gout patients and healthy people. They found that opportunistic pathogens such as Anaerobic bacteria, *Bacteroides* and *Porphyromonas* were significantly increased in gout patients (140). Animal studies have shown that under the effects of *Proteobacteria*, the intestinal nutritional conditions of gout rats are deteriorated, and the permeability of intestinal epithelium and renal endothelium is significantly increased, which promotes the entry of lipopolysaccharide into the blood circulation and significantly aggravates the attack of gout (141). Another study found that compared with normal mice, hyperuricemia mice had significantly less *Bifidobacteria* and *Lactobacilli*, while serum xanthine oxidase activity and lipopolysaccharide levels increased. After treatment with probiotics, beneficial bacteria in the intestinal tract increased, and the expression levels of serum lipopolysaccharide and xanthine oxidase decreased significantly (142). This meta-analysis showed that serum uric acid decreased in patients after probiotic intervention. Yamanaka et al., 2019 (61) and Kamatani et al., 2018 (62) used yogurt containing *L. delbrueckii* ssp. *bulgaricus* and *Streptococcus thermophilus* to treat hyperuricemia and gout, but did not show an improvement in serum uric acid, suggesting that the efficacy of these probiotics needs to be further explored, which provides a reference for screening effective microbiota. Furthermore, since only 4 RCTs were involved, and the quality of the literature was degraded because the details of random sequence generation methods, allocation concealment methods, and blinding methods in some RCTs was not reported, the results need to be interpreted with caution. In addition, those RCTs did not report adverse events, hence, more RCTs reporting efficacy and safety are needed to further explore the treatment of psoriasis with probiotics.

### 4.5 Probiotics for OA

OA is a highly prevalent and disabling disease, affecting more than 7% of the world’s population (about 528 million people), of which knee OA (KOA) accounts for about 85% (141, 143). It is estimated that the prevalence of KOA is about 10% in men and 13% in women among the elderly aged 60 years or older (144). Global life expectancy, changes in dietary habits and lifestyles, aging and obesity prevalence and other social trends have increased the prevalence of KOA, so attention should be paid to the prevention and treatment of KOA (145). In recent years, gut microbes have gradually become a focus of attention in joint inflammatory diseases, and gut microbial imbalance is closely related to the development of OA (146–148). With the progress of microbiome research, modern medicine proposed the concept of “gut-joint” axis. That is, the intestines and joints are closely connected, and diseases of the intestines may induce lesions in the joints. On the contrary, joint diseases can also affect the intestines, and the two are interrelated (149). Gut microbes can positively regulate the host’s immune system, exert immunomodulatory effects (150), protect from pathogenic microorganisms (151), and maintain normal physiological functions. Modulation of dysbiosis and metabolic dysfunction through microbial supplementation may prevent the development of knee injuries (152). For example, prebiotics can reduce systemic inflammatory response and protect articular cartilage in a rodent model of KOA by positively altering the gut microbiota (153). During monoiodoacetate (MIA)-induced KOA, administration of probiotics significantly reduced the expression of proinflammatory cytokines in rat knee articular cartilage (154). In a model of HFS diet-induced knee injury in obese rats, both prebiotic fiber supplementation and aerobic exercise both alone and in combination therapy improved knee injury (153). In a guinea pig model of spontaneous OA, oral administration of *B. longum* CBi0703 significantly improved cartilage structure and exhibited comprehensive joint protection (155). Therefore, controlling the gut microbiota is considered to be a feasible therapeutic strategy to improve obesity-related OA disease (156). Animal studies have shown that chondroitin sulfate combined with probiotics can prevent oxidative stress in the serum of rats with experimental osteoarthritis (157). Probiotics prevent cartilage damage and osteoarthritis progression in mice (158). Only one RCT reported probiotics for OA in this research. Lei et al., 2017 treated 215 patients with *L. casei* Shirota and another 218 with placebo. They found that after 6 months of treatment, compared with the placebo group, patients in the probiotic group had significantly improved WOMAC and VAS scores, and decreased serum hs-CRP levels (P<0.05). They also claim that no serious adverse events were observed throughout the study.

### 4.6 Probiotics for JIA

JIA, defined as arthritis of unknown etiology with onset under the age of 16 and lasting more than 6 weeks, is the most common chronic rheumatic disease in childhood (159–161). At present, more and more studies believe that the pathogenesis of JIA is the result of multiple factors. A genetic susceptible individual is exposed to one or more environmental factors, resulting in local tissue damage, autoantigen release, excessive activation of mononuclear macrophages, neutrophils and other phagocytes, and release of a large number of proinflammatory cytokines, such as IL-1, IL-6, IL-18 and proinflammatory protein S100, which eventually lead to chronic synovitis and systemic multisystem inflammation (162–165). In recent years, the role of gut microbes in regulating the homeostasis of the host has attracted much attention (166). It is gradually recognized that gut microbes may be an important environmental factor involved in the occurrence of JIA and affect disease progression (167). Tejesvi et al. found that the abundance of *Firmicutes* and *Bacteroidetes* increased in the gut microbiota of children with primary, untreated JIA (168). With the advancement of experimental technology, the research on gut microbiota has gradually deepened, and more and more studies have found that the gut microbiome structure characteristics of JIA children are significantly different from those of normal children. It can be manifested as changes in microbial richness and diversity, abnormal composition and structure of some bacterial groups, and a decrease in the number of some beneficial microbiota, such as butyrate-producing bacteria (169). These studies suggest that alterations in the structural characteristics of gut microbes are closely related to the occurrence of JIA. Some scholars have found that the abundance of *Faecalibacterium* in the intestinal microbiota of children with JIA (polyarticular JIA and ERA) is decreased (170). *Faecalibacterium* is a butyrate producing bacteria (BPB), and its reduction can induce an inflammatory state in the body (167). Due to the difficult treatment of JIA at present, the disease is easy to repeat, the long-term application of glucocorticoids and immunosuppressive agents has serious side effects, and the biological preparations are expensive (160, 171). Therefore, it is urgent for the clinic to summarize the efficacy and safety of the intervention effect of probiotic-based microecological preparations in children with JIA.

Most of the probiotics involved in the included studies were *Bifidobacterium, Lactobacillus, Enterococcus* and *Bacillus*, and the doses involved were mostly above 1*10^8 CFU. It is suggested that the intestinal microbiota preparations based on *Bifidobacterium, Lactobacillus, Enterococcus* and *Bacillus* may have better effect when the dose is above 1*10^8 CFU.

### 4.7 Strength and limitations and inspiration for future research

The strength is that this systematic review and meta-analysis evaluated the efficacy and safety of probiotics on 8 types of inflammatory arthritis for the first time, and provided clinical reference.

The limitations of this review are that: (1) The quality of the included RCTs is generally degraded by the lack of detailed random sequence generation, allocation concealment, and blinding information. (2) Vadell et al., 2020 (41) and Lambert et al., 2017 (53) used food containing probiotics and did not give specific strains and doses, while for some of other RCTs, although strains were given, the dosage was uncertain, which further brought heterogeneity. (3) In different diseases, although the same efficacy indicators have been reported, the methods of recording are different. As in psoriasis, Navarro-López et al., 2019 (44) reported the percentage of PASI improvement, but did not provide its specific value, while Lu 2017 (46) and Moludi et al., 2021 (47) reported the specific value of PASI, therefore Navarro-López et al., 2019 (44) was not included in the meta-analysis. (4) Furthermore, most diseases contain fewer than 10 RCTs, and OA even contains only 1 RCT. And only Osteoporosis and Osteopenia included more than 1000 participants (1156), the rest of the diseases included less than 1000 participants. (5) This systematic review only included 8 types of inflammatory arthritis, and the others were not retrieved, which may be due to the fact that probiotics for inflammatory arthritis have just emerged and received less attention. (6) Many RCTs did not report adverse events, perhaps because they were not observed, or were not monitored. These results in the efficacy and safety of probiotics in the treatment of inflammatory arthritis need to be interpreted with caution.

For safety, many RCTs did not report adverse events, raising concerns about the safety of the RCTs included in this review. Based on this, we need to carefully examine many RCTs that do not report adverse events. For example, Tang et al. conducted a search (up to February 2014) of Phase II and IV RCTs of at least one serious adverse event related to drug therapy registered on ClinicalTrials.org, and a total of 1580 RCTs were screened. Then they randomly selected 300 RCTs, and found the corresponding published literature, and compared the agreement between the two serious adverse events, but the results were not completely consistent (172). Specifically, of the 139 RCTs reporting serious adverse events, 44 (32%) RCTs reported a number of serious adverse events in publications that were inconsistent with those recorded on ClinicalTrials.gov. The incidence of serious adverse events reported in the publications of 22 RCTs was lower than the incidence of adverse events recorded on ClinicalTrials.gov, with a difference of more than 30% in each group. Therefore, we consider that the reasons why the included RCTs did not report adverse events may be: (1) RCTs are not registered in the clinical trial center, and the writing is not standardized; (2) There may be cases where only good outcomes are reported, and bad outcomes are not reported; (3) The publications have limited space, etc. This prompts us to interpret the safety results of this study cautiously, and it also suggests that future RCTs should report adverse events in detail. It is also expected that more RCTs in the future can publish the detailed data of the trial in the professional clinical trial database to provide data support for clinical application. In addition, more standardized RCTs reporting on the treatment of other types of inflammatory arthritis with probiotics are needed in the future to further determine the efficacy of probiotics on various inflammatory arthritis.

## 5 Conclusion

Probiotic supplements may improve Hyperuricemia and gout, Inflammatory bowel disease arthritis, JIA, OA, Osteoporosis and Osteopenia, Psoriasis, RA, Spondyloarthritis. However, lack of evidence and heterogeneity of studies do not allow us to recommend them to patients with inflammatory arthritis to manage their disease. More randomized controlled trials are needed in the future to determine the efficacy and optimal dosing design of probiotics.

## Data availability statement

The original contributions presented in the study are included in the article/**Supplementary Material**. Further inquiries can be directed to the corresponding authors.

## Author contributions

LZ and HC are responsible for the study concept and design. LZ, QH, KY, YD, JL, WX, HL, XZ, HC are responsible for the data collection, data analysis and interpretation; LZ and KY drafted the paper; HC supervised the study; all authors participated in the analysis and interpretation of data and approved the final paper.

## Conflict of interest

The authors declare that the research was conducted in the absence of any commercial or financial relationships that could be construed as a potential conflict of interest.

## Publisher’s note

All claims expressed in this article are solely those of the authors and do not necessarily represent those of their affiliated organizations, or those of the publisher, the editors and the reviewers. Any product that may be evaluated in this article, or claim that may be made by its manufacturer, is not guaranteed or endorsed by the publisher.
