# Supplementary material for: Safety and efficacy of probiotic supplementation in 8 types of inflammatory arthritis: A systematic review and meta-analysis of 34 randomized controlled trials
Source: Front Immunol. 2022 Sep 23;13:961325. doi: 10.3389/fimmu.2022.961325 (PMC9547048; doi:10.3389/fimmu.2022.961325)
Supplement: Supplementary file 1 [file Table_1.docx]

**Table S1.** Search Strategies for Pubmed and Embase

| **PubMed** | (probiotic OR synbiotic OR Lactobacillus OR Bifidobacterium OR Streptococcus thermophilus OR Lactococcus OR Bacillus subtilis OR Enterococcus OR Enterococcus faecium OR Enterococcus faecalis OR Saccharomyces OR Probiotics)  AND  ((Arthritis OR Arthritides OR Polyarthritis OR Polyarthritides) OR (Psoriasis OR Psoriases OR Pustulosis of Palms and Soles OR Pustulosis Palmaris et Plantaris OR Palmoplantaris Pustulosis OR Pustular Psoriasis of Palms and Soles) OR (hyperuricemi* OR hyperuricaemi* OR hyperuricacid* OR Gout OR Gouts) OR (Osteoporoses OR Osteoporosis, Post-Traumatic OR Osteoporosis, Post Traumatic OR Post-Traumatic Osteoporoses OR Post-Traumatic Osteoporosis OR Osteoporosis, Senile OR Osteoporoses, Senile OR Senile Osteoporoses OR Osteoporosis, Involutional OR Senile Osteoporosis OR Osteoporosis, Age-Related OR Osteoporosis, Age Related OR Bone Loss, Age-Related OR Age-Related Bone Loss OR Age-Related Bone Losses OR Bone Loss, Age Related OR Bone Losses, Age-Related OR Age-Related Osteoporosis OR Age Related Osteoporosis OR Age-Related Osteoporoses OR Osteoporoses, Age-Related OR Metabolic Bone Diseases OR Bone Disease, Metabolic OR Metabolic Bone Disease OR Osteopenia OR Osteopenias OR Low Bone Density OR Bone Density, Low OR Low Bone Densities OR Low Bone Mineral Density) OR (Rheumatoid arthritis OR Arthritis, Rheumatoid) OR (Osteoarthritis OR Osteoarthritides OR Osteoarthrosis OR Osteoarthroses OR Arthritis, Degenerative OR Arthritides, Degenerative OR Degenerative Arthritides OR Degenerative Arthritis OR Osteoarthrosis Deformans) OR (Spondyloarthritis Ankylopoietica OR Ankylosing Spondylarthritis OR Ankylosing Spondylarthritides OR Spondylarthritides, Ankylosing OR Spondylarthritis, Ankylosing OR Ankylosing Spondylitis OR Spondylarthritis Ankylopoietica OR Bechterew Disease OR Bechterew's Disease OR Bechterews Disease OR Marie-Struempell Disease OR Marie Struempell Disease OR Rheumatoid Spondylitis OR Spondylitis, Rheumatoid OR Spondylitis Ankylopoietica OR Ankylosing Spondyloarthritis OR Ankylosing Spondyloarthritides OR Spondyloarthritides, Ankylosing OR Spondyloarthritis, Ankylosing) OR (Juvenile Arthritis OR Arthritis, Juvenile Chronic OR Chronic Arthritis, Juvenile OR Juvenile Rheumatoid Arthritis OR Arthritis, Juvenile Idiopathic OR Juvenile Chronic Arthritis OR Arthritis, Juvenile Rheumatoid OR Rheumatoid Arthritis, Juvenile OR Juvenile Idiopathic Arthritis OR Idiopathic Arthritis, Juvenile OR Juvenile-Onset Still Disease OR Juvenile Onset Still Disease OR Still's Disease, Juvenile-Onset OR Juvenile-Onset Still's Disease OR Still's Disease, Juvenile Onset OR Still Disease, Juvenile-Onset OR Still Disease, Juvenile Onset OR Systemic Arthritis, Juvenile OR Arthritis, Juvenile Systemic OR Juvenile Systemic Arthritis OR Juvenile-Onset Stills Disease OR Juvenile Onset Stills Disease OR Stills Disease, Juvenile-Onset OR Polyarthritis, Juvenile, Rheumatoid Factor Positive OR Polyarthritis, Juvenile, Rheumatoid Factor Negative OR Oligoarthritis, Juvenile OR Juvenile Oligoarthritis OR Enthesitis-Related Arthritis, Juvenile OR Arthritis, Juvenile Enthesitis-Related OR Enthesitis Related Arthritis, Juvenile OR Juvenile Enthesitis-Related Arthritis OR Psoriatic Arthritis, Juvenile OR Arthritis, Juvenile Psoriatic))  AND  (random* controlled trial [pt] OR controlled clinical trial* [pt] OR randomized [tiab] OR placebo [tiab] OR drug therapy [sh] OR random* [tiab] OR trial* [tiab] OR group* [tiab])  NOT  (animals [mh] NOT humans [mh]) |
| --- | --- |
| **EMBASE** | 1 probiotic*.tw.  2 synbiotic*.tw.  3 'Lactobacillus'/exp  4 lactobacill*.tw.  5 bacill*.tw.  6 'Bifidobacterium'/exp  7 (bifidus or bifidobacter*).tw.  8 'Streptococcus thermophilus'/exp  9 streptococcus thermophilus.tw.  10 streptococc*.tw.  11 'Lactococcus'/exp  12 lactococc*.tw.  13 'Bacillus subtilis'/  14 bacillus subtilis.tw.  15 'Enterococcus'/exp  16 'Enterococcus faecium'/exp or 'Enterococcus faecalis'/  17 'Saccharomyces'/exp  18 saccharomyc*.tw.  19 leuconostoc.tw.  20 pediococc*.tw.  21 bulgarian bacillus.tw.  22 (beneficial adj3 bacter*).tw.  23 (Escherichia coli or "E. coli").tw.  24 Yeast.tw.  25 (fungus or fungi).tw.  26 (VSL# 3 or VSL 3).tw.  27 'Probiotics'/exp  28 or/1-27  29 random$.tw.  30 factorial$.tw.  31 (crossover$ or cross over$ or cross-over$).tw.  32 placebo$.tw.  33 single blind.mp.  34 double blind.mp.  35 triple blind.mp.  36 (singl$ adj blind$).tw.  37 (double$ adj blind$).tw.  38 (tripl$ adj blind$).tw.  39 assign$.tw.  40 allocat$.tw.  41 crossover procedure/  42 double blind procedure/  43 single blind procedure/  44 triple blind procedure/  45 randomized controlled trial/  46 or/29-45  47 Osteoporoses/exp  48 Post-Traumatic Osteoporoses or Post-Traumatic Osteoporosis  49 Senile Osteoporoses or Senile Osteoporosis  50 Age-Related Bone Loss or Age-Related Bone Losses  51 Age-Related Osteoporosis or Age Related Osteoporosis or Age-Related Osteoporoses  52 Osteoarthritis/exp  53 Osteoarthritides/  54 Osteoarthrosis/  55 Osteoarthroses/  56 Degenerative Arthritides/  57 Degenerative Arthritis/  58 Osteoarthrosis Deformans/  59 Spondyloarthritis Ankylopoietica/  60 Ankylosing Spondylarthritis/  61 Ankylosing Spondylarthritides/  62 Ankylosing Spondylitis/  63 Spondylarthritis Ankylopoietica/  64 Bechterew Disease/  65 Bechterews Disease/  66 Marie-Struempell Disease/  67 Marie Struempell Disease/  68 Rheumatoid Spondylitis/  69 Spondylitis Ankylopoietica/  70 Ankylosing Spondyloarthritis/  71 Ankylosing Spondyloarthritides/  72 Arthritis, rheumatoid/exp  73 Rheumatoid arthritis/  74 Arthritis, Juvenile/exp  75 Juvenile Arthritis/  76 Juvenile Rheumatoid Arthritis/  77 Juvenile Chronic Arthritis/  78 Juvenile Idiopathic Arthritis/  79 Juvenile-Onset Still Disease/  80 Juvenile Onset Still Disease/  81 Juvenile Systemic Arthritis/  82 Juvenile-Onset Stills Disease/  83 Juvenile Onset Stills Disease/  84 Juvenile Oligoarthritis/  85 Arthritis, Gouty/exp  86 Gouty Arthritis/  87 Gouty Arthritides/  88 Arthritides, Reactive/exp  89 Reactive Arthritides/  90 Reactive Arthritis/  91 Post-Infectious Arthritides/  92 Post-Infectious Arthritis/  93 Post Infectious Arthritis/  94 Postinfectious Arthritis/  95 Postinfectious Arthritides/  96 Reiter Syndrome/  97 Reiters Disease/  98 Reiters Syndrome/  99 Reiter Disease/  100 Arthritis, Infectious/exp  101 Infectious Arthritis/  102 Viral Arthritis/  103 Bacterial Arthritides/  104 Septic Arthritis/  105 Bacterial Arthritis/  106 Suppurative Arthritis/  107 enteropathic arthritis/  108 traumatic arthritis/  109 Arthritis, Psoriatic/exp  110 Arthritic Psoriasis/  111 Psoriatic Arthritis/  112 Psoriasis Arthropathica/  113 Psoriatic Arthropathy/  114 Psoriatic Arthropathies/  115 Arthritis/exp  116 Arthritides/  117 Polyarthritis/  118 Polyarthritides/  119 47-118/or  120 28 and 46 and 119 |
